# Single‐cell transcriptome atlas revealed bronchoalveolar immune features related to disease severity in pediatric *Mycoplasma pneumoniae* pneumonia

**DOI:** 10.1002/mco2.748

**Published:** 2024-10-13

**Authors:** Xiantao Shen, Zhengjiang Jin, Xiaomin Chen, Zhenhui Wang, Lu Yi, Yangwei Ou, Lin Gong, Chengliang Zhu, Guogang Xu, Yi Wang

**Affiliations:** ^1^ State Key Laboratory of Environment Health (Incubation) Key Laboratory of Environment and Health Ministry of Education Key Laboratory of Environment and Health (Wuhan) Ministry of Environmental Protection School of Public Health Tongji Medical College Huazhong University of Science and Technology Wuhan China; ^2^ Department of Clinical Laboratory Maternal and Child Health Hospital of Hubei Province Tongji Medical College Huazhong University of Science and Technology Wuhan China; ^3^ Department of Disinfection and Pest Control Wuhan Center for Disease Control & Prevention Wuhan China; ^4^ Department of Radiology Maternal and Child Health Hospital of Hubei Province Tongji Medical College Huazhong University of Science and Technology Wuhan China; ^5^ Department of Clinical Laboratory Institute of Translational Medicine Renmin Hospital of Wuhan University Wuhan China; ^6^ Health Management Institute The Second Medical Center & National Clinical Research Center for Geriatric Diseases Chinese PLA General Hospital Beijing China; ^7^ Experimental Research Center Capital Institute of Pediatrics Beijing China

**Keywords:** immune response, *Mycoplasma pneumoniae*, *Mycoplasma pneumoniae* pneumonia, protective immunity, single‐cell sequencing

## Abstract

The mechanisms underlying protective immunity in mild *Mycoplasma pneumoniae* pneumonia (MPP) and the pathogenesis of severe MPP, characterized by dysregulated immune responses, remain unclear. Here, we performed single‐cell RNA sequencing (scRNA‐seq) to profile bronchoalveolar lavage fluid (BALF) samples from 13 healthy donors and 24 hospitalized pediatric patients with MPP, covering both mild and severe cases. Severe MPP patients exhibited high levels of exhausted T cells and M1‐like macrophages, with the exhaustion of T cells attributed to persistent type I interferon signaling and inadequate assistance from CD4^+^ T cells. Significant cell‐cell interactions between exhausted T cells and programmed death‐ligand 1^+^ (PD‐L1^+^) macrophages were detected in severe patients, potentially mediated through inhibitor molecules (e.g., PD1) and their receptors (e.g., PD‐L1), as well as human leukocyte antigen class I molecules and their receptors (e.g., KLRC1/D2), resulting in the dysfunction of anti‐MP immune responses. Mild MPP patients were featured by an increased abundance of neutrophils, coupled with enhanced activation, contributing to protective immunity. Together, our study provides a detailed characterization of the BALF immune landscape in MPP patients, revealing distinct immune characteristics between mild and severe cases, which offers a valuable resource for understanding MPP immunopathogenesis and formulating effective therapeutic strategies.

## Graphical abstract

Severe patients were characterized by high levels of exhausted T cells and M1‐like macrophages, with the exhaustion of T cells attributed to persistent type I interferon signaling and inadequate assistance from CD4^+^ T cells. Significant cell‐cell interactions between exhausted T cells and programmed death‐ligand 1^+^ (PD‐L1^+^) macrophages were detected in severe patients, potentially mediated through inhibitor molecules (e.g., PD1) and their receptors (e.g., PD‐L1), as well as human leukocyte antigen class I molecules and their receptors (e.g., KLRC1/D2), resulting in the dysfunction of anti‐MP immune responses. Mild patients were featured by an increased number of neutrophils, along with a more pronounced level of neutrophil activation, contributing to the development of protective immunity.

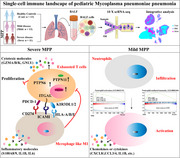

## 1 INTRODUCTION

Since mid‐October 2023, the World Health Organization (WHO) has monitored data from Chinese surveillance systems, indicating an increase in respiratory illness among children, particularly in norther regions such as Beijing (Nov 23, 2023; www.who.int/emergencies/disease‐outbreak‐news).^1^ This pattern mirrors trends in other countries, where common respiratory diseases resurged during the first post‐pandemic winter following the relaxation of measures (e.g., travel restrictions and mask‐wearing). Surveillance data from China suggested that *Mycoplasma pneumoniae* (MP), a common bacterial infection primarily affecting younger children, is a leading cause of this surge.^1^

MP, the etiological agent of *Mycoplasma pneumoniae* pneumonia (MPP), is a significant human pathogen primarily responsible for lower respiratory tract infections.^2^ MP is responsible for 8−40% of community‐acquired pneumonia (CAP) cases in children.^3^ While generally considered self‐limiting and resulting in mild, self‐resolving,^4^ MPP can lead to severe symptoms with prolonged disease progression, lung injury, and even fatal outcomes in some individuals.^5^ In recent years, the increasing prevalence of macrolide‐resistant MP strains has reduced the efficacy of antibiotic treatment in children,^4^ further emphasizing the threat that MP poses to pediatric health. Thus, understanding the pathogenesis of MPP is crucial for identifying therapeutic targets and effectively combating the current epidemic. However, little information is available about the mechanisms for pathogenesis in MP infection.

Many studies have provided valuable insights into the pathogenesis of MP infections. For example, MP is capable of adhering to host cells via its apical structure during infection, causing direct physical damage and nutrient depletion.^6^ Apart from direct cellular damage, an exaggerated immune response is a substantial feature of MPP.^4^ Animal experiments highlighted the crucial role of inflammation in MPP development.^7^ MP infection induces an increase in interleukin (IL)‐8 expression in respiratory epithelial cells, triggering the release of metalloproteinase‐9 (MMP‐9), myeloperoxidase (MPO), and neutrophil elastase (NE).^8^ Furthermore, IL‐17 can trigger autophagy in lung epithelial cells and promote the release of multiple proinflammatory factors (e.g., S100A8/A9) from neutrophils.^9^ In clinical settings, alterations in immune cell frequencies were observed in MPP patients, including increased neutrophil concentrations in bronchoalveolar lavage fluid (BALF) compared to patients with other forms of pneumonia. Although these studies have shed light on the pathogenesis of MP infection, a comprehensive understanding of immune responses to MP infections remains incomplete, necessitating further investigation.

Single‐cell RNA sequencing (scRNA‐seq) has emerged as a powerful tool for unraveling immune responses and has been successfully applied to investigate infectious diseases such as coronavirus disease 2019 (COVID‐19), tuberculosis (TB), and brucellosis.^10^, ^11^, ^12^, ^13^, ^14^ However, a comprehensive understanding of pathogenic immune response in MPP patients remains elusive. scRNA‐seq analysis of immune cells from MPP patients has the potential to provide critical insights into this complex interplay within the lung microenvironment. BALF, a rich source of immune cells reflecting the bronchiolar and alveolar microenvironment, offers a particularly valuable platform for such investigations. Herein, characterizing BALF cells through scRNA‐seq holds promise for deciphering the complex lung responses to MP infection.

To comprehensively and unbiasedly characterize the lung immunological landscape and its association with disease severity in MP‐infected patients, we conducted scRNA‐seq on BALF cells obtained from 37 individuals. This cohort comprised 24 hospitalized MPP patients with varying clinical presentations and 13 healthy controls. This comprehensive single‐cell atlas provides a valuable resource for elucidating the pathogenic immune response in MPP. Moreover, our findings offer crucial insights with implications for developing targeted therapies against MP infection, especially for those with severe disease.

## 2 RESULTS

### 2.1 Single‐cell transcriptional profiling of BALF cells

**FIGURE 1:**
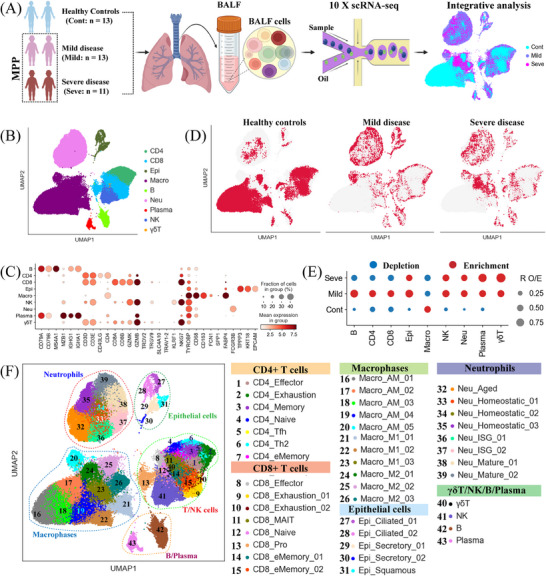
An overview of the results and showcases of the study design for bronchoalveolar lavage fluid (BALF) single‐cell transcriptomic study. (A) Diagram outlining the overall study design. Thirty‐seven samples were obtained from 37 individuals, including 24 *Mycoplasma pneumoniae* pneumonia (MPP) patients (13 patients with mild disease and 11 patients with severe disease) and 13 healthy donors. (B) The clustering result (Left row) of nine major cell types (right row) from 37 samples. Each point represents one single cell, colored according to cell type. (C) Dot plots of the nine major cell types (Columns) and their marker genes (Rows). (D) Uniform manifold approximation and projection (UMAP) of the healthy controls, mild and severe conditions. (E) Disease preference of major cell clusters as estimated using the ratio of observed to expected cell counts (R_O/E_). (F). The clustering result (Left row) of 43 cell subtypes (right row) from 37 samples. Each point represents one single cell, colored according to cell type.

To uncover the immunological characteristics of patients with MPP, we employed droplet‐based scRNA‐seq (10xGenomics) to analyze the transcriptomic profiles of BALF cells from 24 MPP patients, encompassing a spectrum of disease severity from mild to severe, and 13 healthy individuals. Single‐cell transcriptional profiles for healthy controls were obtained from a previously published study.^15^ Based on these data, we classified the 37 participants into three clinical conditions: mild (Mild, *n* = 13), severe (Seve, *n *= 11), and healthy controls (Cont, *n* = 13) (Figure 1A). The clinical characteristics and laboratory findings of the 24 MP‐infected patients included in the study are detailed in Table S1 and Figure S1. Particularly, the severe and mild groups were comparable in age, with no statistically significant difference observed (Figure S2). A total of 156,781 cells were obtained from the 37 samples. After performing computational doublet removal, 101,683 cells successfully passed quality control (QC; see methods) (Figure S2A–C). These included 47,696 cells from 13 mild patients, 17,130 cells from severe patients, and 36,857 cells from healthy controls (Figure S2D). Each BALF sample yielded an average of 2748 cells (Figure S2A–D).

Following clustering analysis, we manually annotated nine major cell types based on the expression of canonical marker genes and variable genes, namely CD4^+^ T cells (CD4), CD8^+^ T cells (CD8), plasma B cells, B cells, natural killer cells (NK cells), γδ T cells, epithelial cells (Epi), neutrophils (Neu), and macrophages (Macro) (Figure 1B,C and Table S2). Further sub‐clustering analysis revealed an additional 43 cell subtypes (Figure 1F and Figure S2E–I). Our analysis identified clusters unique to our dataset, including a proliferating B cell subtype (plasma B cell) and distinct subpopulations of macrophages and neutrophils (Figure 1 and Figure S2). The identified cell lineages (*n* = 9) and sub‐lineages (*n* = 43) in this study represent a wide range of cell types in BALF samples, providing a rich resource for precise annotation and analysis of BALF cells at different levels of resolution.

Uniform manifold approximation and projection (UMAP) analysis revealed the recognizable differences among disease status (Figure 1D and Figure S3). Major cell clusters and subclusters exhibited varying degrees of enrichment corresponding to disease severity (Figures 1, 3, 4, 5, and 6B). To unveil distinctive immune profiles across different disease conditions, we conducted an analysis of immune cell composition using R_O/E_ (the ratio of observed to randomly expected cell numbers used for removing the technical variations on tissue preference estimation), a method employed to mitigate technical variations in tissue preference estimation, as described in previous reports.^10^, ^12^ Notably, mild MPP patients exhibited a higher enrichment of B, CD4^+^ T, and CD8^+^ T cells (Figure 1E, Figure S2K, and Table S3). The frequencies of three lymphocyte types (plasma B cells, NK cells, and γδ T cells) and the epithelial cell cluster were elevated in MPP patients, while plasma B cells and γδ T cells seemed to be more enriched in severe MPP patients (Figure 1E, Figure S2K, and Table S3). Within myeloid cells, the proportions of neutrophils were increased in MPP patients, aligning with previous findings that neutrophils were the primary immune cells involved in lung infiltration,^4^, ^16^ thereby confirming the accuracy and reliability of our scRNA‐seq analysis. Unlike neutrophils, which increased in BALF, macrophages were obviously decreased in MPP patients (Figure 1E, Figure S2K, and Table S3). Furthermore, ANOVA analysis demonstrated associations between each major cluster and different patient groups (Figure S2J). Interestingly, myeloid cells (e.g., macrophages) from BALFs displayed an obvious association with severe MPP patients, suggesting a potential role for these myeloid cells in the progression of severe MMP. In contrast, most lymphocytes (e.g., CD4^+^ T, CD8^+^ T, plasma B cells, and NK cells) were evidently associated with mild MMP. Together, these findings suggested that each disease status in MP‐infected patients may be characterized by a specific immune signature.

### 2.2 Pro‐inflammatory cytokines in MPP primarily originated from myeloid cells

**FIGURE 2:**
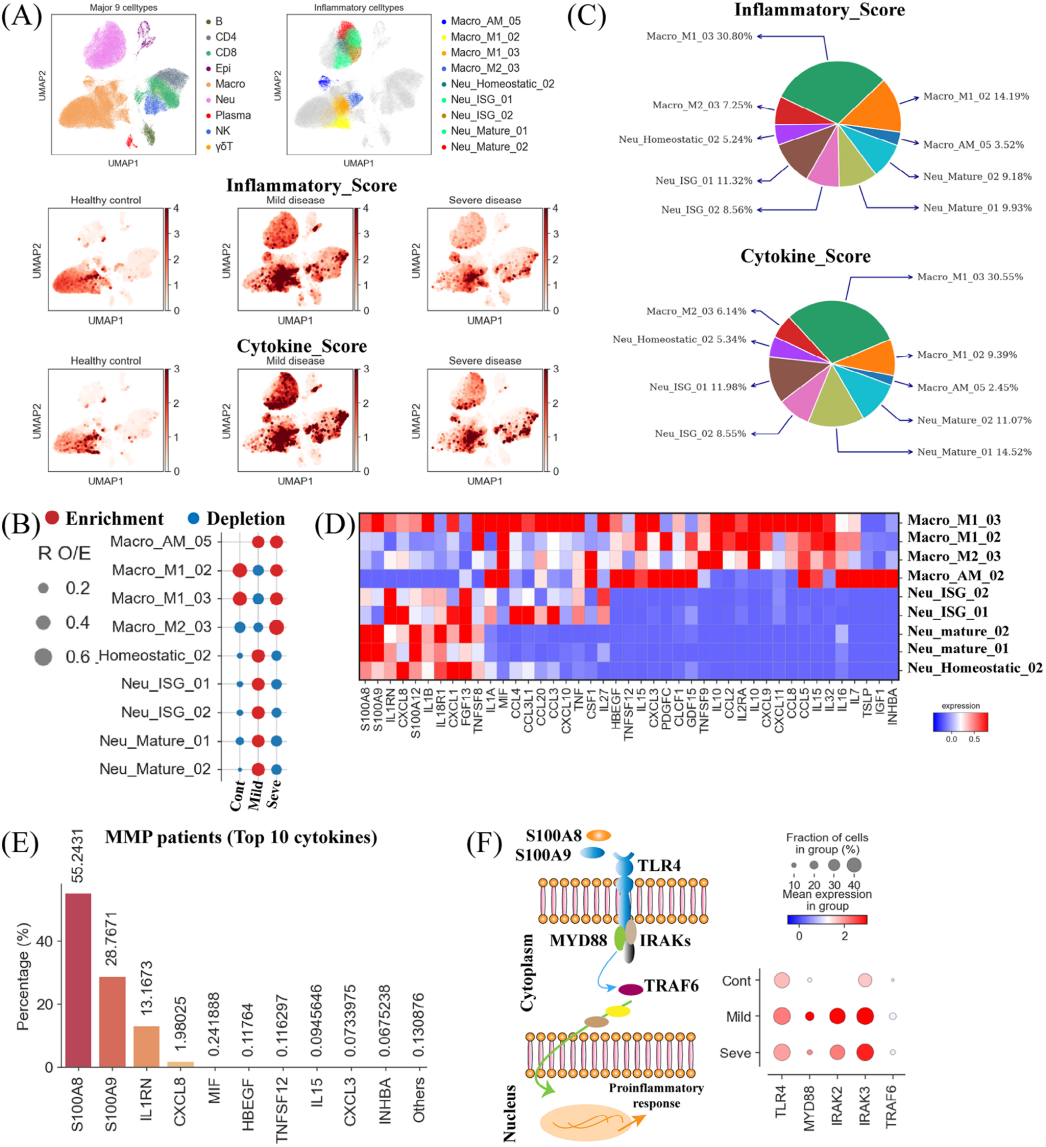
Myeloid cells are the primary contributors to the production of pro‐inflammatory cytokines in *Mycoplasma pneumoniae* pneumonia (MPP). (A) Uniform manifold approximation and projection (UMAP) of bronchoalveolar lavage fluids (BALFs). Colored based on the nine major cell types (top left), nine hyper‐inflammatory cell subtypes (top right), inflammatory (Middle), and cytokine score (Bottom). (B) Disease preference of nine hyper‐inflammatory cell subsets as estimated using the ratio of observed to expected cell counts (R_O/E_). (C) Pie charts depicting the relative contribution of each inflammatory cell subtype to the cytokine and inflammatory scores. (D) Heatmap depicting the expression of cytokines within each hyper‐inflammatory cell subtype identified. (E) Bar chart depicting the relative contribution of the top 10 cytokines in MPP patients. (F) The expression analysis of *S100A8*/*A9*‐*TLR4*‐*MyD88* pathway.

The inflammatory response plays a significant role in the development of MPP,^7^ while the origins of cytokine production at the infection site in MPP patients remain unclear. To investigate the potential sources of cytokine genes, we systematically analyzed the expression of established inflammatory response genes and a curated set of cytokine genes (Table S4).^10^, ^12^ We calculated an inflammatory score and a cytokine score for each cell based on the expression levels of these genes, using these scores as metrics to assess the potential role of each cell in the inflammatory response in MP‐infected patients. Our analysis revealed a noticeable increase in the expression of inflammatory and cytokine genes in MPP patients compared to healthy controls (Figure 2A), suggesting the presence of an inflammatory response. However, severe MPP patients did not exhibit significantly higher expression levels of inflammatory and cytokine genes compared to those with mild disease (Figure 2A). Further analysis of our scRNA‐seq data identified significantly higher inflammatory and cytokine scores in nine cell subtypes, including four macrophage subtypes (Macro_AM_05, Macro_M1_02, Macro_M1_03, and Macro_M2_03) and five neutrophil subtypes (Neu_Homeostatic_02, Neu_ISG_01, Neu_ISG_02, Neu_Mature_01, and Neu_Mature_02), indicating that these cells may be major contributors to the inflammatory response and the primary sources of pro‐inflammatory cytokines (Figure 2A and Figure S2). Interestingly, MPP patients exhibited significantly higher expression levels of inflammatory and cytokine genes in nine inflammatory cell clusters compared to healthy controls (Figure S5A), further validating our findings that macrophages and neutrophils are likely the major contributors to the inflammatory response in MPP patients.

We next investigated the frequencies of nine cell subsets in MP patients. At the bulk level, we observed a significant enrichment of these inflammatory cell subsets in individuals with MP infection (Figure S5B). The distribution of inflammatory cell subsets within BALF exhibited varying enrichment patterns among disease states (Figure 2B). In mild disease, there was a notable enrichment of inflammatory neutrophils, whereas inflammatory macrophages seemed to be more prevalent in severe cases (Figure 2B). Approximately 70% of the inflammatory and cytokine scores in MPP patients were attributable to five specific inflammatory cell subtypes, namely Macro_M1_03, Macro_M1_02, Neu_mature_01, Neu_Mature_02, and Meu_ISG_01, highlighting their crucial involvement in the inflammatory response (Figure 2C). Certain lymphocyte subsets (e.g., CD4_Exhaustion), along with myeloid cells, might also contribute to the inflammatory response in MPP patients through the elevated expression of pro‐inflammatory cytokines (Figure S4). These findings suggested that inflammatory cell subtypes, especially Macro_M1_03, Macro_M1_02, Neu_mature_01, Neu_Mature_02, and Meu_ISG_01, might play a central role in driving the lung inflammatory response in MPP patients, as indicated by their high expression of inflammatory and cytokine genes as well as increased cell frequencies.

Next, we sought to characterize the inflammatory signatures specific to each subset of inflammatory cells. Each inflammatory cell subset showed a distinct expression profile of inflammatory cytokine genes (Figure 2D), suggesting diverse mechanisms underlying the inflammatory response in MPP patients. Notably, in MPP patients, inflammatory macrophage subsets exhibited pronounced expression of pro‐inflammatory cytokines (e.g., *S100A8/A9/A12*, *CXCL8*, *CLCF1*, *MIF*, *CCL8*, *IL10*, etc.) (Figure 2D). Likewise, inflammatory neutrophil subtypes displayed significant upregulation of specific pro‐inflammatory cytokines (e.g., *IL1RN*, *CXCL1*, *IL1B*, *FGF13*, *S100A8/A9/A12*, etc.) (Figure 2D). We identified the top 10 most highly expressed proinflammatory cytokines, including *S100A8*, *S100A9*, *IL1RN*, *CXCL8*, *MIF*, *HBEGF*, *TNFSF12*, *IL15*, *CXCL3*, and *INHBA*, collectively contributed to approximately 99% of the total cytokine scores in MPP patients, indicating their central involvement in the inflammatory response (Figure 2E). Four of these pro‐inflammatory molecules, including *S100A8*, *S100A9*, *IL1RN*, and *CXCL8*, are primarily secreted by inflammatory macrophages and neutrophils, and these four alone accounted for approximately 98% of the total cytokine scores, suggesting a central role for these specific molecules in driving the inflammatory response in MPP (Figure 2E and Figure S5C). The expression of *S100A8*, *S100A9*, *IL1RN*, and *CXCL8* genes was significantly upregulated in MPP patients (Figure S5D), further corroborating the findings of our scRNA‐seq data analysis. In addition to these top 10 pro‐inflammatory cytokines, we also detected high expression of other typical pro‐inflammatory cytokine gens like *S100A12*, *CCL2/3/4/5/8*, *TNF*, *CSF1*, etc. (Figure S5E), further supporting the involvement of diverse mechanisms in eliciting the inflammatory response in MPP patients. Despite not being the primary drivers of the inflammatory response (Figure 2 and Figure S5E), the upregulation of these additional cytokines likely contributes to the amplification and perpetuation of the inflammatory process.

Among the top 4 pro‐inflammatory cytokines identified, *S100A8* and *S100A9* emerged as potential central mediators in triggering the inflammatory response in MPP patients, as these two molecules collectively accounted for approximately 85% of the total cytokine score. (Figure 2E). *S100A8/9*, highly expressed during inflammation, is known to modulate inflammation by triggering cytokine release and promoting leukocyte recruitment.^17^ *S100A8/9* complex predominantly binds to *TLR4* (toll‐like receptor 4), and upon activation, this interaction triggers the release of a wide array of pro‐inflammatory cytokines, thereby intensifying the inflammatory cascade (Figure 2F).^17^, ^18^ Consistent with this, our analysis revealed that *TLR4* is predominantly expressed in macrophages and neutrophils (Figure S5F). Activation of the *S100A8/9*‐*TLR4* signaling axis engages the MyD88‐dependent pathway, characterized by the recruitment and activation of interleukin‐1 receptor‐associated kinases (IRAKs; e.g., *IRAK1* and *IRAK3*) and TNF receptor‐associated factor 6 (*TRAF6*) (Figure 2F), ultimately leading to amplification of the inflammatory response and potential lung damage.^17^ Indeed, we observed significantly increased expression of essential genes within the *S100A8/9*‐*TLR4*‐*MyD88* signaling pathway in TLR4‐expressing cells from MPP patients (Figure 2F). These results highlight the prominent role of *S100A8/9*‐*TLR4*‐driven inflammatory signaling in MPP and underscore the potential of targeting pro‐inflammatory S100A8/A9 proteins as a therapeutic strategy to mitigate immunopathogenesis in MPP patients. In addition to S100A8/A9, the upregulated expression of other inflammatory cytokines (e.g., *CXCL8*, *S100A12*, *CCL2/3/4/5*, etc.) likely contributes to the amplification of the inflammatory response. For instance, *IL‐8* (*CXCL8*) can induce the secretion of MPO, NE, and MMP‐9.^8^

To further investigate the systemic inflammatory response in MPP, we measured the plasma concentrations of 34 cytokines in MPP patients and healthy donors (Cohort 2) (Figure S6 and Table S5). Compared to healthy donors, MPP patients did not exhibit a significant increase in plasma levels of pro‐inflammatory cytokines (e.g., IL‐1B, IL‐6, IL‐10, IL‐8, IP‐10, IL‐15, IL‐1RA, MIP‐1α/β, MCP‐1, etc.) (Figure S6). These data suggested that peripheral immune cells may not contribute significantly to the inflammatory response observed in MMP patients, highlighting the localized nature of inflammation within the lung microenvironment.

### 2.3 Exhausted CD8^+^ T cells as a potential cause of severe MPP

**FIGURE 3:**
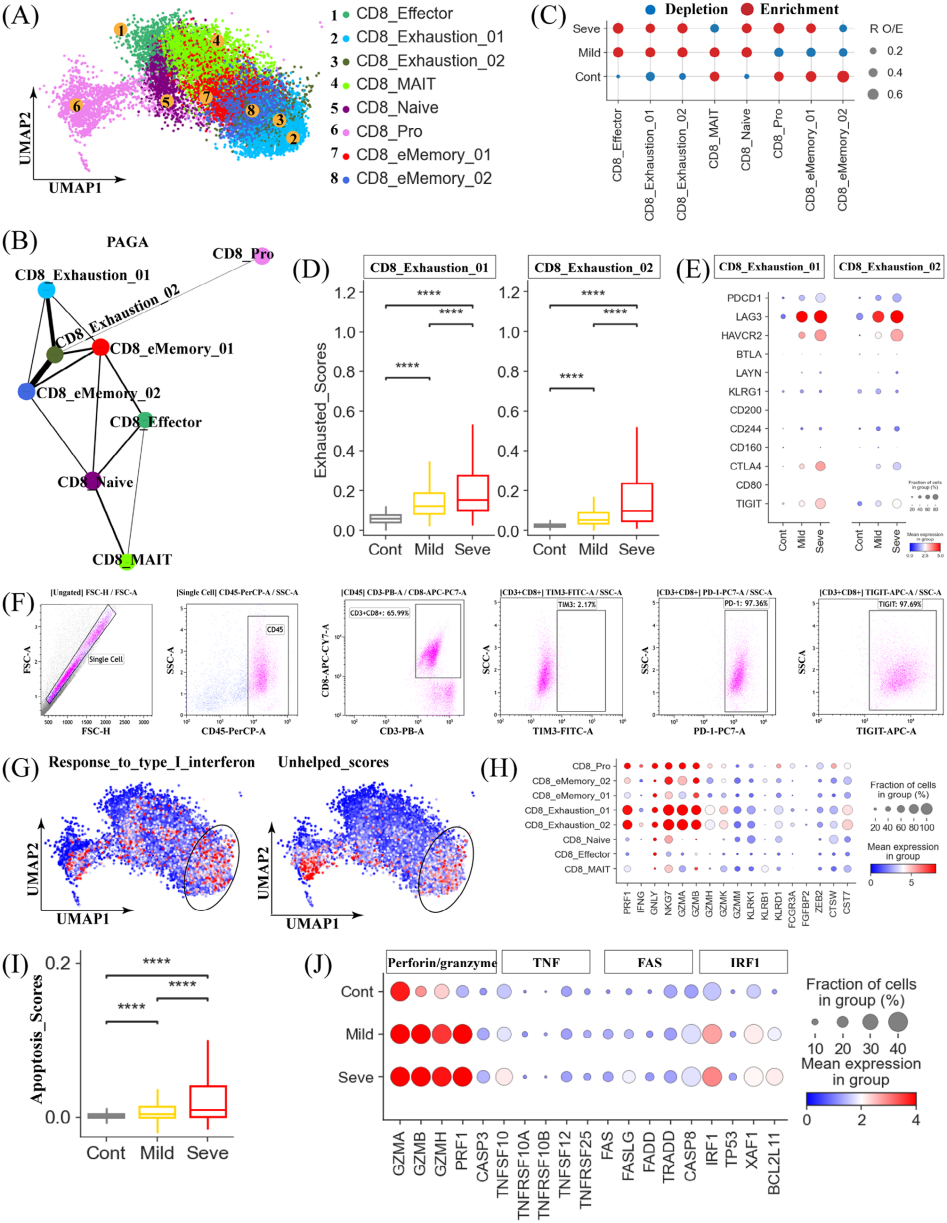
Immunological features of CD8^+^ T‐cell subsets. (A) The clustering result (Left row) of 8 CD8^+^T cell types (right row) from 37 samples. Each point represents one single cell, colored according to cell type. (B) PAGA analysis of CD8^+^ T cell pseudo‐time: the associated cell type and the corresponding status are listed. (C) Disease preference of CD8^+^ T cell clusters as estimated using R_O/E_. (D) Box plots showing the exhausted scores in CD8_Exhausted_01/02 subsets across disease conditions. Significance was evaluated using the Kruskal‐Wallis test with Bonferroni correction (*p < 0.05, **p < 0.01, ***p < 0.001, ****p < 0.0001, and ^ns^ *p* > 0.05). (E) Dot plots showing the cell exhaustion‐related markers in CD8_Exhausted_01/02 subsets across disease conditions. (F) Flow cytometry plots showing gating strategy and typical exhausted molecules in CD8^+^T cells from severe cases (Cohort 2‐Sample 1). (G) Uniform manifold approximation and projections (UMAPs) illustrating IFN‐I response and unhelped signature scores for each CD8^+^T cell. (H) Dot plots showing the cytotoxicity‐related genes in CD8^+^T cell subsets. (I) Box plots showing the apoptosis scores in CD8^+^T cells across disease conditions. Significance was evaluated using the Kruskal‐Wallis test with Bonferroni correction (*p < 0.05, **p < 0.01, ***p < 0.001, ****p < 0.0001, and ^ns^ *p* > 0.05). (J) Dot plots showing the apoptosis‐related genes in CD8^+^T cell subsets.

Clustering analysis further stratified the CD8^+^ T cell population into 8 distinct subclusters, including naïve (CD8_Naive), effector (CD8_Effector), effector memory (CD8_eMemory_01 and CD8_eMemory_02), exhausted (CD8_Exhaustion_01 and CD8_Exhaustion_02), mucosal‐associated invariant T (MAIT) cells (CD8_MAIT), and a proliferating subcluster (CD8_Pro) (Figure 3A and Figure S1). Analysis of previously identified signature genes (Table S6) revealed different functional statuses for each CD8^+^T cell subcluster. As expected, CD8_Naive cells exhibited the highest naïve scores in CD8_Naive, while CD8_Exhaustion_01, CD8_Exhaustion_02, and CD8_Pro cells displayed high exhaustion scores. Notably, CD8_Exhaustion_01 and CD8_Exhaustion_02 also exhibited high inflammatory and cytotoxic scores (Figure S7A).

To delineate the potential developmental transitions of CD8^+^ T cell subsets, we employed PAGA (partition‐based graph abstraction) analysis to reconstruct the developmental trajectories of the eight identified CD8^+^ T cell subtypes (Figure 3B). As anticipated, CD8T_MAIT cells formed a distinct branch, supporting their classification as MAIT cells. Among the remaining CD8^+^ T cell subtypes, CD8_Pro and CD8_Exhaustion_01 cells formed two different branches. Interestingly, the PAGA map unveiled multiple nodes with high connectivity between different CD8^+^ T cell subtypes, suggesting potential trans‐differentiation pathways. Specifically, CD8_eMemory_01 and CD8_eMemroy_02 cells appeared to represent an intermediate state, connecting naïve CD8^+^ T cells (CD8_Naive) to activated CD8^+^ T‐cell subsets (e.g., CD8_Exhasution_01 and CD8_Exhasution_02). We observed particularly high connectivity between CD8_Exhasution_01 and CD8_Exhasution_02 cells, as well as CD8_eMemroy_02 and CD8_Exhasution_02 cells.

Analysis of the eight CD8^+^ T cell clusters revealed disease‐specific heterogeneities (Figure 3C and Figure S7B). We observed a noticeable increase in the proportion of the MAIT cells in MPP patients with mild disease (Figure 3C), implying that a MAIT cell response might contribute to the control of MP infection. Notably, the proportions of both exhausted CD8^+^ T‐cell subtypes (CD8_Exhaustion_01 and CD8_Exhaustion_02) were increased in MPP patients compared to healthy controls (Figure 3C and Figure S7B). These exhausted CD8^+^ T clusters also exhibited significantly higher exhaustion scores, with the highest exhaustion scores observed in patients with severe disease (Figure 3D). These findings indicated that MP infection can induce CD8^+^ T cell exhaustion, which may contribute to the impaired anti‐MP immune response observed in critically ill patients. Consistently, CD8_Exhaustion_01 and CD8_Exhaustion_02 cells from patients with severe disease expressed higher levels of multiple exhaustion‐associated genes (e.g., *PDCD1*, *LAG3*, *HAVCR2*, *CTLA4*, etc.) compared to cells from healthy controls and mild patients (Figure 3E).

*HAVCR2* (*Tim‐3*) interacts with galectin‐9, *BTLA* with *TNFRSF14* (also known as *HVEM*), and *PDCD1* with *PD‐L1*/*PD‐L2*. These interactions result in the recruitment of the tyrosine‐protein phosphatase *SHP1* (also known as *PTPN6*) and/or *SHP2* (also known as *PTPN11*) via immunoreceptor tyrosine‐based switch motifs (ITSMs) or immunoreceptor tyrosine‐based inhibitory motifs (ITIMs) within their intracellular domains.^12^ This signaling cascade ultimately inhibits LAT‐Zap70 and PI3K‐AKT signaling pathways, leading to decreased cell proliferation and cytokine production. In addition, increased expression of *PRDM1* has been connected to elevated inhibitory receptor expression and reduced polyfunctionality in exhausted cells.^12^ As expected, the expression levels of these core transcriptional regulators (e.g., *PTPN6/11* and *PRDM1*) were noticeably higher in exhausted CD8^+^T cells from MP‐infected individuals, particularly those with severe disease, compared to healthy donors (Figure S7C). These data further support our findings that MP infection, particularly those with severe disease, can induce CD8^+^ T cell exhaustion. Flow cytometry analysis confirmed the exhausted phenotypes of CD8^+^ T cells in patients with severe disease (Figure 3F and Figure S8A). T cell exhaustion can severely compromise the ability of the immune system to effectively control infections, thus indicating that exhausted CD8^+^ T cells likely contribute to the severity of MPP.

Previous studies established a direct link between the development of the exhaustion program and persistent type I interferon (IFN) signaling.^19^ Consistent with this, we observed robust enrichment of type I IFN signaling‐associated genes in exhausted CD8^+^ T cells, suggesting a direct link between the exhausted phenotype of CD8_Exhaustion_01 and CD8_Exhaustion_02 cells and persistent type I IFN signaling (Figure 3G and Figure S7D). In addition, CD8^+^ T cell exhaustion has been strongly associated with impaired CD4^+^ T cell help, a crucial process by which CD4^+^T cells support the differentiation and maintenance of robust CD8^+^ memory T cell responses during infection.^19^ Given the dependence of robust CD8^+^ memory T cell generation on CD4^+^ T cell help, we investigated whether the exhausted CD8^+^ T cell subtypes (CD8_Exhaustion_01 and CD8_Exhaustion_02) exhibited transcriptional signatures indicative of insufficient CD4^+^ T cell help. As anticipated, CD8_Exhaustion_01 and CD8_Exhaustion_02 displayed an obvious enrichment of transcripts specifically associated with unhelp CD8^+^ T cells (Figure 3G and Figure S7D). These results indicated that the exhaustion phenotype observed in CD8_Exhaustion_01 and CD8_Exhaustion_02 cells is likely driven by a combination of persistent type I IFN signaling and insufficient CD4^+^ T cell help.

Despite showing characteristics of unhelped and exhausted T cells, CD8_Exhaustion_01 and CD8_Exhaustion_02 cells exhibited an obvious enrichment of cytotoxicity signature genes (Figure S8B) and higher expression levels of cytotoxicity‐associated genes like *PRF1*, *GNLY*, *NKG7*, *GZMA*/*B*/*H*/*K* and *KLRK1*, etc., revealing the potential heterogeneity within these two exhausted subtypes (Figure 3H). This observation is consistent with previous functional studies demonstrating that, while exhausted CD8^+^ T cells exhibit impaired proliferative capacity and cytokine production, their cytotoxic functions remain largely intact.^19^, ^20^ MPP patients, particularly those with severe symptoms, showed enhanced expression of cytotoxicity‐related signature genes in CD8_Exhaustion_01 and CD8_Exhaustion_02 cells compared to healthy controls (Figure S8B). While the cytolytic functions of effector CD8^+^ T cells are essential for eliminating pathogens and infected cells through the release of cytotoxic granules containing perforin, granzymes, and granulysin, these effector molecules can also contribute to immunopathology by promoting inflammation and extracellular matrix degradation.^12^ Therefore, the augmented expression of multiple cytolytic molecules in CD8^+^ T cells might contribute to immunopathology in severe MPP patients.

In addition to their role in promoting cytotoxic functions, cytolytic molecules (e.g., granzymes and perforins) also trigger cell apoptosis.^21^ We, therefore, investigated apoptosis‐related profiles in CD8^+^T cells from MPP patients. Using an apoptosis scoring system, we found that CD8^+^T cells at the bulk level were more prone to apoptosis in severe MPP patients compared to both healthy controls and patients with mild disease (Figure 3I). Likewise, severe patients displayed significantly higher apoptosis scores across the majority of CD8^+^ T cell subsets (Figure S8C). We next analyzed the expression of signature genes related to apoptosis‐related pathways, including perforin/granzyme, *FAS*, *TNF*, and *IRF1* pathways.^12^ We observed an upregulated trend in the expression of genes within *TNF*, *FAS*, and *XAF1* pathways (*GZMB*, *TNFSF10*, *FASLG*, and *IRF1*) in CD8^+^T cell subsets from MPP patients (Figure 3I), particularly those with severe disease. However, the expression of certain apoptosis‐related genes (*TNFRSF10A*, *TP53*, and *FADD*) was not significantly different between groups. These findings suggested that the increased expression of genes involved in the perforin/granzyme, *TNF*, *FAS*, and *XAF1* apoptosis pathways may potentially result in increased apoptosis of CD8^+^T cell subsets in MPP patients, specifically in those with severe disease.

For the CD8^+^T cells, we also identified a proliferative subset (CD8_Pro), characterized by high expression of *MKI67* and *TYMS*, as well as multiple effector genes (e.g., *NKG7*, *GZMA*, *GZMH*, *CST7*, etc.). This expression profile confirms this subset as a proliferative effector memory CD8^+^ T cell population (Figure S8D). PAGA analysis revealed that CD8_Pro primarily originated from CD8_Exhaustion_02 cells (Figure 3C). Particularly, the increased presence of CD8_Pro cells and their precursor cells in severe MP patients may indicate a partial dysregulation of CD8^+^ T cell‐mediated adaptive immunity (Figure 3B). Similar to exhausted CD8^+^T cells, CD8_Pro cells also exhibited elevated levels of exhaustion, cytotoxicity, and apoptosis markers, particularly in patients with severe MPP (Figure S8E), potentially further exacerbating the dysregulated CD8^+^ T cell response.

### 2.4 Expansion of exhausted CD4^+^T cells in MPP patients

**FIGURE 4:**
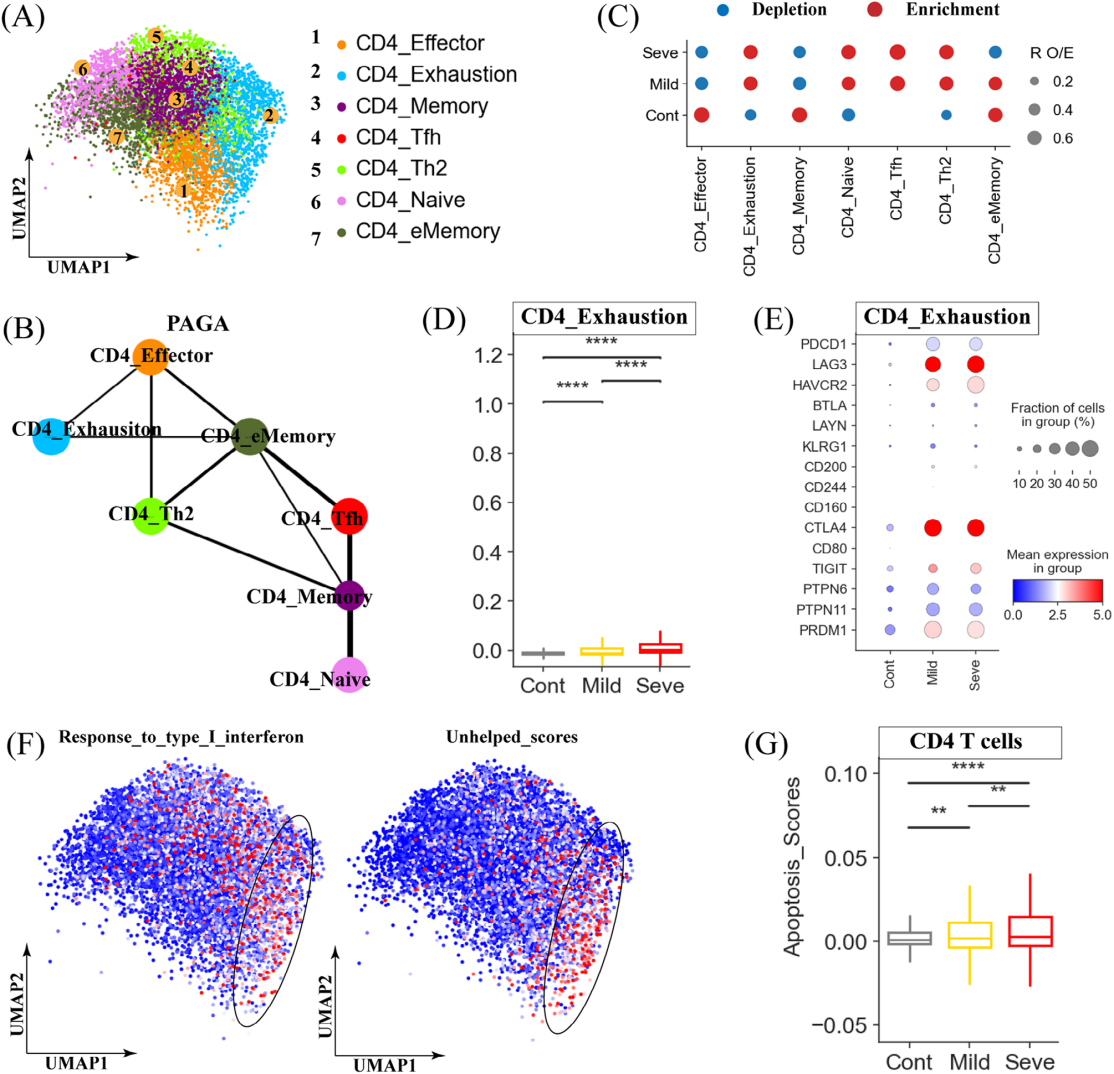
Immunological features of CD4^+^ T‐cell subsets. (A) The clustering result (Left row) of 7 CD4^+^T cell types (right row) from 37 samples. Each point represents one single cell, colored according to cell type. (B) PAGA analysis of CD4^+^ T cell pseudo‐time: the associated cell type and the corresponding status are listed. (C) Disease preference of CD4^+^ T cell clusters as estimated using the ratio of observed to expected cell counts (RO/E). (D) Box plots showing the exhausted scores in the CD4_Exhaustion subset across disease conditions. Significance was evaluated using the Kruskal‐Wallis test with Bonferroni correction (*p < 0.05, **p < 0.01, ***p < 0.001, ****p < 0.0001, and ^ns^ *p* > 0.05). (E) Dot plots showing the cell exhaustion‐related genes in the CD4_Exhaustion subset across disease conditions. (F) Uniform manifold approximation and projections (UMAPs) illustrating IFN‐I response and unhelped signature scores for each CD4^+^T cell. (G) Box plots showing the apoptosis scores in CD4^+^T cells across disease conditions. Significance was evaluated using the Kruskal‐Wallis test with Bonferroni correction (*p < 0.05, **p < 0.01, ***p < 0.001, ****p < 0.0001, and ^ns^ *p* > 0.05).

We identified seven distinct CD4^+^ T cell clusters: naïve (CD4_Naive), memory (CD4_Memory), effector (CD4_Effector), effector memory (CD4_eMemroy), follicular helper T (Tfh) cells (CD4_Tfh), Th2 (CD4_Th2) and exhausted CD4^+^T cells (CD4_Exhaustion) (Figure 4A and Figure S2). PAGA trajectory analysis unveiled three distinct developmental trajectories for CD4^+^ T cells, culminating in CD4_Exhaustion, CD4_Th2, and CD4_Effector cells as different endpoints (Figure 4B). The developmental trajectory correlated with the functional scores of the different cell types, with CD4_Exhaustion cells exhibiting the highest exhaustion scores and CD4_Effector cells displaying the highest cytotoxic scores (Figure S9A). Moreover, CD4_Exhaustion cells exhibited a heightened inflammatory state, whereas CD4_Naive cells displayed high naïve scores (Figure S9A).

We next examined the proportions of CD4^+^ T cell subsets across different disease states (Figure 4C and Figure S9B). MPP patients exhibited an increased abundance of CD4_Tfh cells compared to healthy controls (Figure 4C). CD4_Tfh cells displayed a transcriptional profile indicative of Tfh cells, including high expression of *ICOS* and *MAP* (Figure S2), suggesting their potential involvement in promoting protective B cell immune responses. In addition to CD4_Tfh, three other subsets, including CD4_Naive, CD4_Th2, and CD4_Exhaustion, were also enriched in MPP patients (Figure 4C). Interestingly, exhausted CD4^+^ T cells (CD4_Exhaustion) share transcriptional similarities with exhausted CD8^+^ T cells, including high expression of inhibitor molecules (e.g., *PDCD1*, *LAG3*, *CTLA4*, and *HAVCR2*) and exhaustion‐related transcriptional factors like *PTPN6*/*11* and *PRDM1* (Figure S10A). Importantly, the degree of CD4^+^ T cell exhaustion appeared to be greater in patients with severe MPP, indicating a more profound state of exhaustion in these individuals (Figure 4D,E). Further analysis revealed that similar to exhausted CD8^+^ T cells, the exhausted phenotype observed in the CD4_Exhaustion cluster was linked to persistent type I IFN signaling and potentially inadequate CD4^+^ T cell help (Figure 4E and Figure S10B). These findings suggest that MP infection can drive CD4^+^ T cell exhaustion, which may contribute to the dysregulated anti‐MP infection response, particularly in severe MPP.

Consistent with our observations of CD8^+^ T cells, CD4^+^ T cells also displayed heightened apoptosis markers, particularly in patients with severe MPP (Figure 4G). The primary apoptotic mechanisms implicated in CD4^+^ T cell apoptosis included the perforin/granzyme, *TNF*, *FAS*, and *XAF1* pathways (Figure S10C). We found that three CD4^+^T subsets, including CD4_Exhaustion, CD4_Effector, and CD4_Th2, were particularly susceptible to apoptosis (Figure S10D).

### 2.5 Infiltration and activation of neutrophils in mild MPP

**FIGURE 5:**
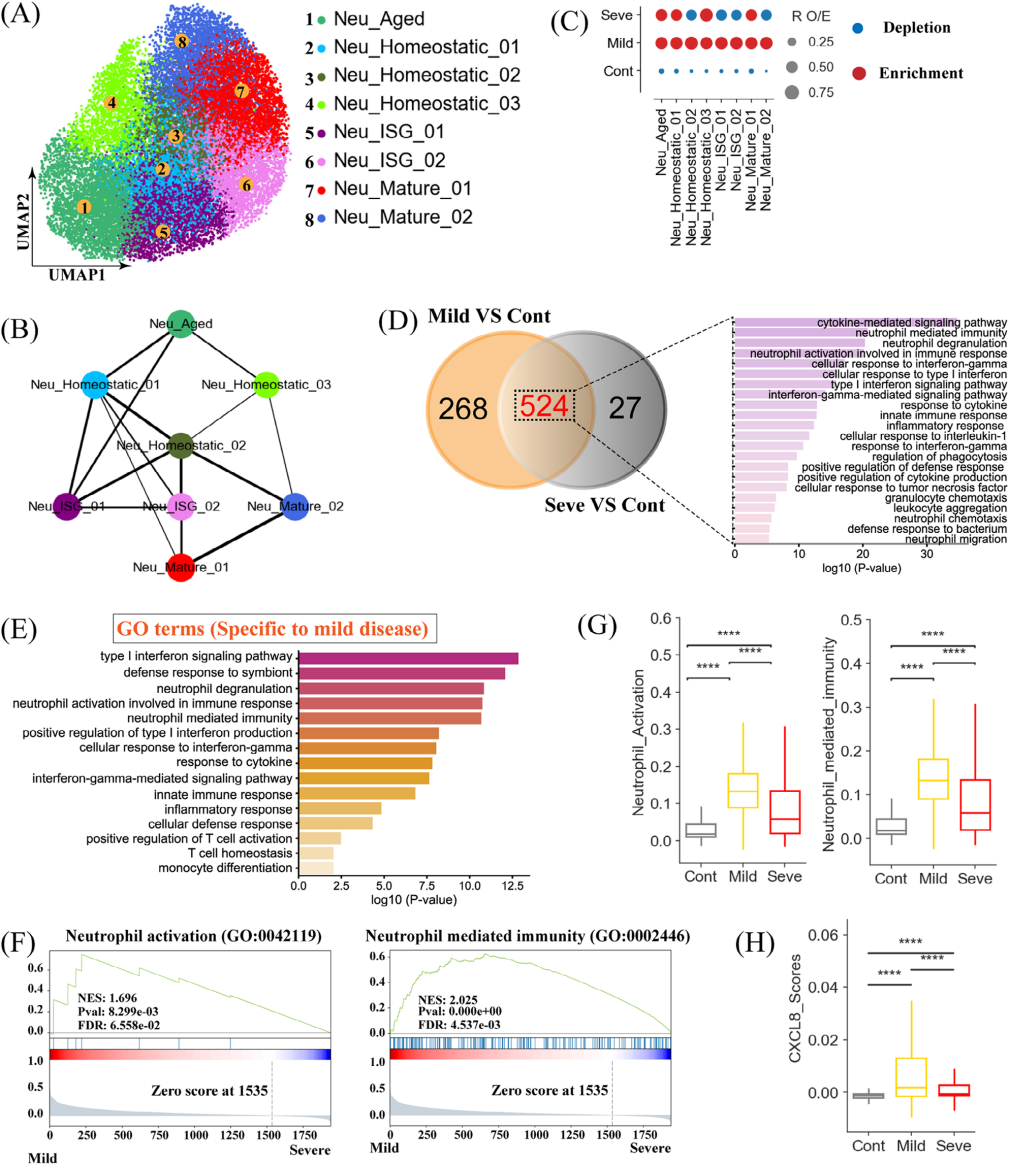
Immunological features of neutrophils. (A) The clustering result (Left row) of neutrophil subtypes (right row) from 37 samples. Each point represents one single cell, colored according to cell type. (B) PAGA analysis of neutrophil pseudo‐time: the associated cell type and the corresponding status are listed. (C) Disease preference of neutrophil clusters as estimated using the ratio of observed to expected cell counts (R_O/E_). (D) Venn diagram illustrating the number of upregulated genes in neutrophils. (E) Enriched Gene Ontology (GO) biological process terms for upregulated genes in neutrophils from mild disease. Only select terms are shown. (F) Gene Set Enrichment Analysis of the sets compared between mild and severe groups with the neutrophil activation and neutrophil‐mediated immunity gene sets. (G) Box plots showing the indicated scores in neutrophils across disease conditions. Significance was evaluated using the Kruskal‐Wallis test with Bonferroni correction (*p < 0.05, **p < 0.01, ***p < 0.001, ****p < 0.0001, and ^ns^ *p* > 0.05). (H) Box plots showing the expression scores of CXCL8 in neutrophils across disease conditions. Significance was evaluated using the Kruskal‐Wallis test with Bonferroni correction (*p < 0.05, **p < 0.01, ***p < 0.001, ****p < 0.0001, and ^ns^ *p* > 0.05).

Sub‐clustering analysis unveiled substantial heterogeneity within the neutrophil compartment, identifying eight distinct subtypes (Figure 5A). To annotate these neutrophil subsets, we utilized marker gene signatures from published reports,^22^, ^23^ identifying two mature subsets (Neu_Mature_01 and Neu_Mature_02), three homeostatic subsets (Neu_Homeostatic_01, Neu_Homeostatic_02, and Neu_Homeostatic_03), two ISG (interferon‐stimulated genes)‐related subsets (Neu_ISG_01 and Neu_ISG_02), and one aged subset (Neu_Aged_01) (Figure 5A and Figure S2). We then employed PAGA to reconstruct the trajectory of neutrophil state transitions (Figure 5B). PAGA analysis uncovered multiple nodes with high connectivity between different neutrophil subtypes, suggesting potential trans‐differentiation pathways. The mature cluster (Neu_Mature_01) displayed a strong transition towards ISG‐related neutrophils (Neu_ISG_02) (Figure 5B). Notably, the Neu_Homeostatic_01 subset appeared to represent an intermediate state, connecting with most other neutrophil subtypes (Figure 5B), highlighting its potential as a target for therapeutic intervention.

All eight neutrophil subsets were present in both healthy controls and MPP patients, while their proportions differed significantly depending on the disease severity (Figure 5C). Notably, all neutrophil subsets were more abundant in MPP patients compared to healthy controls (Figure 5C), with the most pronounced changes observed in patients with mild disease (Figure S11A). Specifically, we observed a significant enrichment of two ISG‐related neutrophil subsets (Neu_ISG_01 and Neu_ISG_02) in patients with mild disease. These subsets were characterized by elevated expression of ISG‐related genes like *ISG15*, *ISG20*, *MX1*, etc. (Figure 5C). In addition to ISGs, ISG‐related neutrophils also expressed high levels of calgranulins (*S100A8*/*A9*/*A12*), cytokines (*IL1B*), and chemokines (*CXCL8*, *CCL3*, and *CCL4*), contributing to the modulation of inflammation (Figure S11B). Interestingly, several other neutrophil subsets, including Neu_Mature_01, Neu_Mature_02, Neu_Homeostatic_01, Neu_Homeostatic_02, and Neu_Aged_01, also exhibited pro‐inflammatory characteristics, expressing high levels of cytokines (e.g., *IL1B*), chemokines (e.g., *CXCL8*), and calgranulins (e.g., *S100A8*) (Figure S11B). These findings suggest that neutrophil expansion, particularly in mild disease, may be a hallmark of MPP, potentially correlating with disease severity.

To further investigate functional differences between mild and severe MPP, we performed differentially expressed genes (DEGs) analysis of neutrophils. We identified 792 and 551 upregulated genes in neutrophils from mild and severe MPP patients, respectively, compared to healthy controls. Among these, 524 genes were commonly upregulated genes in both disease conditions (Figure 5D). Gene Ontology (GO) enrichment analysis revealed that these shared upregulated genes was enriched for terms related to neutrophil function and immune response, including ‘neutrophil‐mediated immunity’, ‘neutrophil activation involved in immune response’, ‘defense response to bacterium’, and ‘neutrophil migration’, consistent with the established role of neutrophils in fungal and bacterial infections.^24^ GO terms related to ‘inflammatory response’, ‘response to cytokine’, and ‘positive regulation of cytokine production’ were also enriched in MPP patients (Figure 5D), indicating that neutrophils from MPP patients exhibit pro‐inflammatory characteristics. Interestingly, these commonly upregulated genes were also enriched for terms related to IFN responses, including ‘cellular response to interferon‐gamma’ and ‘type I interferon signaling’, etc. (Figure 5D). While IFN‐γ and IFN‐I responses are typically associated with intracellular bacterium and viral infection, respectively,^10^ their roles in the context of MPP warranted further investigation.

We next focused our analysis on the 268 upregulated DEGs specifically enriched in mild MPP (Figure 5D), as these genes might contribute to protective immunity. GO enrichment analysis revealed that these upregulated genes were enriched for terms related to neutrophil activation, including “neutrophil activation involved in immune response”, “neutrophil‐mediated immunity” and “cellular defense response”, et, al. (Figure 5E), suggesting that neutrophil activation may be more pronounced in mild MPP compared to severe MPP. Consistent with this, pathway analysis revealed a significant enrichment of DEGs involved in neutrophil activation pathways in mild MPP patients (Figure 5F and Figure S11C). Additionally, we observed an enrichment of DEGs involved in neutrophil chemotaxis and migration in mild MPP patients (Figure S11C). Notably, neutrophil activation and neutrophil‐mediated immunity scores were substantially higher in mild MPP patients (Figure 5G), particularly within the Nue_ISG_01 and Neu_homeostatic_02 subsets (Figure S11D). Consistently, key genes (e.g., CXCL8) involved in neutrophil activation were also upregulated in mild MPP (Figure 5H and Figure S11E). CXCL8 (IL‐8), a potent neutrophil chemoattractant and activator,^4^ was expressed at lower levels in severe MPP, suggesting that impaired CXCL8‐mediated neutrophil activation may contribute to disease severity and highlighting its potential as a therapeutic target in severe cases. Collectively, these findings indicated that enhanced neutrophil recruitment and activation in mild MPP may contribute to protective immunity against MP infection.

### 2.6 Enrichment of suppressive‐like macrophages in severe MPP

**FIGURE 6:**
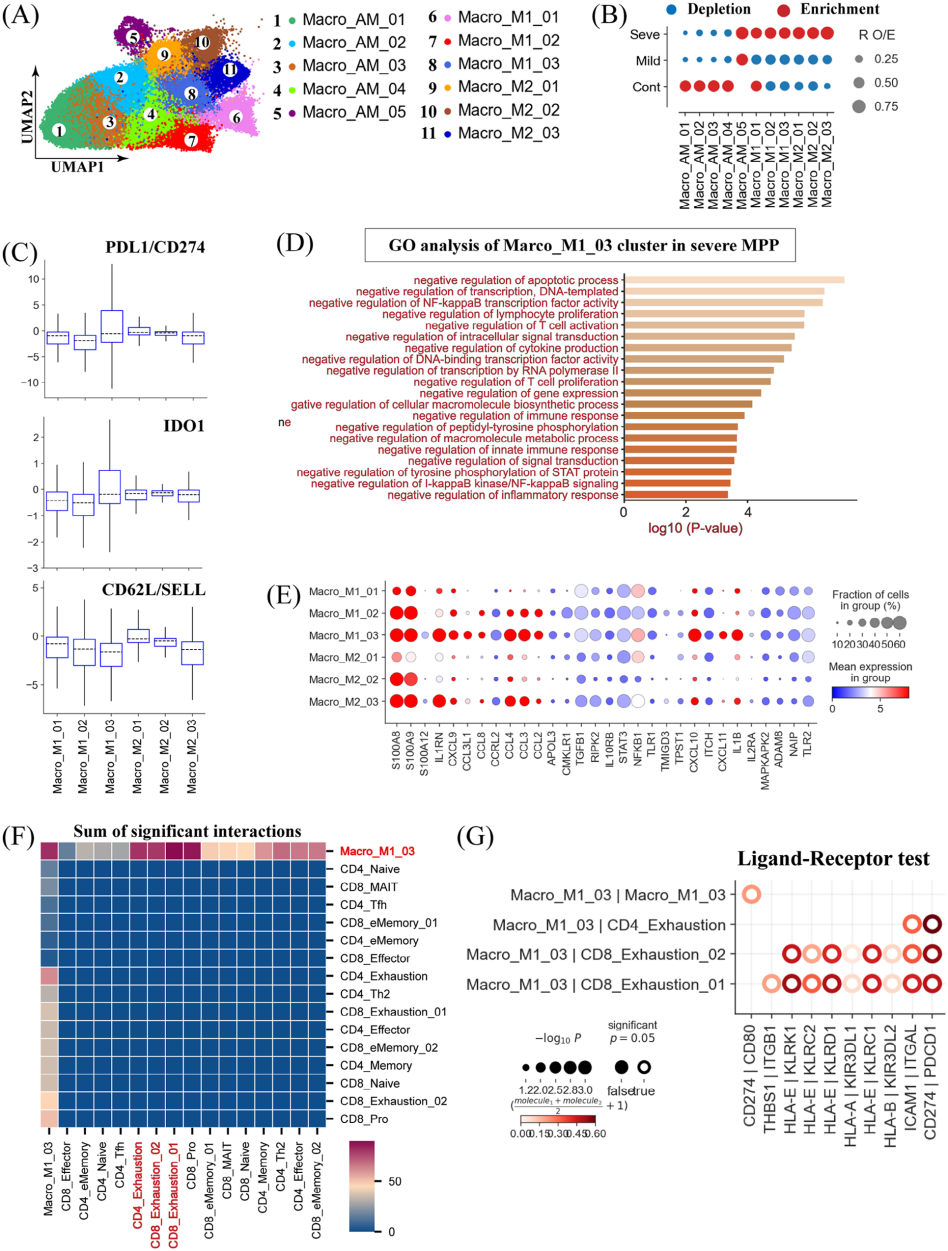
Immunological features of macrophages. (A The clustering result (Left row) of macrophage subtypes (right row) from 37 samples. Each point represents one single cell, colored according to cell type. (B) Disease preference of macrophage clusters estimated using the ratio of observed to expected cell counts (R_O/E_). (C) Box plots showing the expression scores of PD‐L1, SELL, and IDO1 in M1‐ and M2‐like macrophages. (D) Enriched Gene Ontology (GO) biological process terms for upregulated genes in Macro_M1_03 from severe disease. Only select terms are shown. (E) Dot plots showing the inflammation‐related genes in M1‐ and M2‐like macrophages.

We obtained 50705 macrophages across the three disease conditions and observed significant remodeling of macrophage populations between patients with mild and severe MPP (Figure 6A‐B and Figure S12A‐B). To further dissect macrophage heterogeneity, we re‐clustered these cells into 11 subtypes (Figure 6A‐B and Figure S2). Based on established classification criteria and commonly used markers,^25^, ^26^ we grouped macrophages according to their *MRC1*, *MARCO*, *FCN1*, *SPP1* and *FABP4* expression patterns into three major groups: alveolar‐like macrophages (Macro_AM_01/02/03/04/05), classical monocyte‐like macrophages (Macro_M1_01/02/03) and M2‐like macrophages (Macro_M2_01/02/03) (Figure 6A and Figure S2). In healthy lung tissue, the AM‐like population accounted for over 80% of the total macrophage population (Figure S12C). However, MPP patients exhibited a significant depletion of most AM‐like macrophage subsets (Figure 6B). In contrast, M1‐ and M2‐like macrophages comprised approximately 80% of the total macrophage population in the lungs of MPP patients (Figure S12C). Most M1‐ and M2‐like macrophage subtypes were enriched in MPP patients, particularly those with severe symptoms (Figure 6B).

Given their pronounced remodeling in MPP, particularly in severe cases (Figure 6B and Figure S12A,B), we further characterized the M1‐ and M2‐like macrophages with more granularity. We identified a macrophage cluster (Macro_M1_03), charactered by low *SELL* (*CD62L*) expression and high *PD‐L1* (*CD274*) and *IDO1* expression, that was strongly associated with severe MPP (Figure 6C and Figure S12A,B). High *PD‐L1* expression and *CD62L* downregulation have been frequently linked to suppressive functions in neutrophil, granulocytic, monocytic, and macrophagic myeloid‐derived suppressor cells (MDSCs).^27^, ^28^ In addition, *IDO1* ^+^ macrophages have been implicated in immunosuppressive functions in the context of lung tuberculosis.^26^ These observations suggested that the Macro_M1_03 cluster in severe MPP highly resembled macrophagic MDSCs (M‐MDSCs) (Figure 6C). Supporting this notion, Macro_M1_03 cells from patients with severe MPP exhibited high *PD‐L1* expression and low CD62L expression (Figure S12D), further confirming Macro_M1_03 as M‐MDSCs. These findings suggested a potential immunosuppressive role for Macro_M1_03 cells in severe MPP.

To further explore the functional characteristics of Macro_M1_03 cells, we performed GO enrichment analysis on DEGs of Macro_M1_03 cells between MPP patients and healthy controls. Genes associated with immunosuppressive functions, including ‘negative regulation of T cell activation/proliferation’, ‘negative regulation of immune response and innate immune response’, ‘negative regulation of intracellular signal transduction’, etc., were significantly enriched in Macro_M1_03 cells from patients with severe MPP (Figure 6D). These results are consistent with the known immunosuppressive properties of MDSCs and suggest that Macro_M1_03 may suppress T cell activation and function in severe MPP, potentially impairing the adaptive immune response against MP.^10^, ^12^ Consistent with this hypothesis, we observed increased expression of multiple immunosuppressive genes (e.g., *CD86*, *IDO1*, *CD80*, *HAVCR2*, etc.) (Figure S12E). In addition to their immunosuppressive phenotype, Macro_M1_03 cells also exhibited a pro‐inflammatory phenotype, expressing high levels of various inflammatory genes (e.g., *CCL2/3/4/8*, *S100A8/9/12*, and *IL1B*, etc.) (Figure 6E). These findings suggest that Macro_M1_03 cells in severe MPP displayed a complex phenotype characterized by both immunosuppressive and pro‐inflammatory features.

To investigate the potential crosstalk of Macro_M1_03 and T cells, we performed cell‐cell interaction analysis. Interestingly, Macro_M1_03 cells exhibited preferential interactions with exhausted T cells (e.g., CD8_Exhaustion_01/02 and CD4_Exhaustion) in severe MPP (Figure 6F). We further explored the ligand‐receptor (L‐R) pairs mediating these interactions (Figure 6G). We identified multiple L‐R pairs between HLA class I molecules (HLA‐I; e.g., HLA‐A/B/E) and killer cell lectin‐like receptor family (KLR; e.g., KLRC1/C2) with high interaction potentials (Figure 6G). In addition, several inhibitor receptor‐ligand pairs, including PDCD1‐CD274/PD‐L1, also displayed strong potential interaction potentials (Figure 6G). The HLA‐E_KLRD1 axis has been shown to play a crucial role in mediating T‐cell dysfunction and viral persistence during chronic hepatitis B virus (HBV) and hepatitis C virus (HCV) infections.^29^ Moreover, the interaction between PDCD‐1 on T cells and PD‐L1 on macrophages inhibits T cell activation, contributing to immune evasion by tumors or pathogens.^30^ Collectively, these findings offer a framework for future studies investigating the contribution of macrophage‐T cell interactions to MPP pathogenesis and the development of novel therapeutic strategies targeting these interactions.

## 3 DISCUSSION

In this report, we applied scRNA‐seq to create a detailed immune landscape of the lung immune microenvironment at a single‐cell resolution in pediatric patients with varying degrees of MPP severity. These findings enhance our understanding of MPP pathogenesis across a spectrum of disease presentations and may facilitate the identification of novel immune targets or the development of innovative therapeutic strategies for the effective management of severe disease.

Our comprehensive analysis offers an unbiased characterization of the immunological landscape in pediatric patients with MP infection (Figures 1, 2, 3, 4, 5, 6). Mild MPP was characterized by an expansion of neutrophils and a corresponding increase in neutrophil activation. These findings provide valuable insights into the early immune response against MP, particularly in patients presenting with mild symptoms. The observed neutrophilia in mild MPP is in line with the established role of neutrophils as primary responders in the innate defense against respiratory pathogens, including MP.^31^ As essential components of the innate immune system, their increased presence in mild cases signifies a prompt and targeted response against the invading MP. Moreover, significant neutrophil activation in these cases suggests an enhanced state of readiness and functionality (Figure 5). This heightened immune activity in mild MPP may contribute to the effective clearance of MP, potentially limiting the infection's severity and duration. Hence, the specific immunological signatures observed in mild cases could be of great importance in tailoring therapeutic interventions and advancing MP infection management strategies. Although these findings provide valuable insights into the immunological landscape for neutrophils in mild MPP cases, further research is warranted to unravel the intricate molecular mechanisms underlying the observed neutrophil response.

Unlike mild MPP patients who exhibit neutrophilia, severe MPP patients are characterized by an elevation of exhausted T cells, revealing a distinctive aspect of the immune response in this cohort. This exhausted T cell phenotype in severe MPP patients suggests a state of functional impairment, hindering their ability to effectively combat the pathogen. This observation may provide insight into the impaired pathogen control observed in these individuals. Our study firstly validated the presence of T cell exhaustion in severe MPP patients, substantiated by (i) increased expression of multiple inhibitory molecules (e.g., PD‐1, LAG3, and Tim‐3, etc.) and (ii) heightened expression of exhaustion‐related transcription factors (TFs; e.g., *PTPN6*). The augmented expression of inhibitory receptors and exhaustion‐related TFs illuminates the mechanistic basis for compromised T cell functionality in severe MPP patients. For instance, PD‐1 engaging with PD‐L1/PD‐L2, and Tim‐3 with galectin‐9 initiates the recruitment of PTPN6 and/or PTPN11 through their intracellular domains. This recruitment inhibits key signaling pathways like LAT‐Zap70 and PI3K‐AKT, ultimately resulting in decreased T‐cell proliferation, activation, and cytokine production.^12^ Therefore, understanding these specific molecular mechanisms lays the groundwork for developing targeted interventions that aim to modulate inhibitory pathways and transcriptional regulation, ultimately restoring T cell functionality and improving the immune response in severe MPP.

In addition to increased exhausted T cells, severe MPP patients also exhibited an increase in macrophagic MDSCs (M‐MDSCs) (Figure 6). The elevated abundance of M‐MDSCs in these patients may signify a broader immunosuppressive environment, likely contributing to disease severity. Particularly, this dual immune dysregulation, including exhausted T cell and M‐MDSCs responses, presents widespread dysfunction in both adaptive and innate immune components. This dysfunction potentially contributes to the challenges faced by severe MPP patients in controlling the infection. In our study, we conclusively identified a distinct M‐MDSCs (Macro_M1_03) featured decreased SELL expression and increased PD‐L1 and IDO1 expression. The inverse correlation between CD62L (encoded by *SELL*) and PD‐L1 aligns with the established patterns associated with the suppressive functions of MDSCs.^27^ These data provide a further understanding of the immunosuppressive mechanisms driving severe MPP. Moreover, IDO1‐expressing macrophages, known for their suppressive functions in pulmonary tuberculosis,^26^ provide a parallel reference to other respiratory infections like MPP, highlighting the conservation of these suppressive mechanisms across diverse pulmonary conditions. Collectively, these unique traits displayed by M‐MDSCs in severe cases suggest their potential involvement in modulating the immune response during severe MPP, contributing to our understanding of disease pathogenesis.

Interestingly, our findings support the hypothesis that M‐MDSCs (Macro_M1_03) may directly impair T cell activation and function (Figure 6). T cells, which are crucial for orchestrating immune responses against pathogens like MP, occupy an important position within the adaptive immune system.^32^ Consequently, inhibition of T cells by Macro_M1_03 could compromise the host's ability to mount an efficient immune response in severe MPP. Therefore, we next investigated the potential crosstalk between Macro_M1_03 and T cells to uncover the mechanisms underlying this dysregulated response. Our analysis revealed that, in severe cases, Macro_M1_03 exhibited a dominant interaction with exhausted T cells (Figure 6). Multiple L‐R pairs, including HLA‐I molecule (e.g., HLA‐A/B/E) with killer cell lectin‐like receptor family (e.g., KLRC1/C2), and inhibitor receptors (e.g., PD‐1) with their ligands (e.g., PD‐L1), showed significant interaction potential (Figure 6). Recent findings on the HLA‐E_KLRD1 axis, which mediates T‐cell dysfunction and viral persistence in chronic HBV/HCV infections,^29^ exhibit intriguing parallels to our observed dysregulated immune responses in severe MPP. This connection implies that similar immune modulation pathways may be active in diverse infection scenarios, emphasizing the broader significance of our findings. In addition, the interaction between PD‐1 on exhausted T cells and PD‐L1 on M‐MDSCs, identified in our study, provides a critical mechanistic insight into the suppression of T cell activation during severe MPP.^33^ This observation adds another layer to the complex immune evasion tactics employed by MP. Consequently, immune checkpoint inhibitors targeting PD‐1 or PD‐L1 might represent a potential strategy to enhance the immune response against persistent MP in severe cases. Together, a comprehensive understanding of the interactions between exhausted T cells and M‐MDSCs could provide valuable insights for developing therapeutic strategies to modulate the immune response during MP infection. Such strategies could potentially improve pathogen clearance while minimizing immunopathology.

Interpretation of this study may be constrained by several limitations. Despite analyzing a relatively large number of cells, the study included only 24 MPP patients. This relatively small sample size, particularly when divided into mild and severe groups, might limit the statistical power and generalizability of the findings. Our study analyzed BALF samples collected at a single time point during the acute stage of MPP. Herein, this study provides a snapshot of the immune response but does not capture the dynamic changes in immune cell populations and their functions over the course of the disease. Our exclusively focused on BALF, which reflects the immune microenvironment of the bronchoalveolar space. While this is a relevant site for MP infection, it might not fully represent the systemic immune response to MPP. In addition, while our study utilized a publicly available dataset of healthy children for comparison, potentially introducing racial bias, the comparison between our severe and mild MPP groups, both consisting of children of the same racial background, strengthens the validity of our findings regarding the immunological pathogenesis of MPP, especially for patients with severe MMP.

In summary, our study presented a comprehensive analysis of the lung immune cell landscape across different disease severities of MP infection. Despite the limitation of a relatively small sample size, our data provides a valuable resource for deepening our understanding of immune responses in MP infection. Furthermore, our findings offer valuable insights for the development of innovative immunotherapeutic strategies for MPP, especially for severe cases.

## 4 METHODS

### 4.1 Ethical approval

The study was conducted in accordance with the Declaration of Helsinki and received ethical approval from the Ethics Committee of Maternal and Child Health Hospital of Hubei Province (Ethical approval NO.2023IEC122).

### 4.2 Study design and participants

This study included pediatric patients diagnosed with MPP admitted to the Maternal and Child Health Hospital of Hubei Province between October 12 and November 6, 2023. Cases were classified as mild MPP and severe MPP according to the Guidelines for Diagnosis and Treatment of MPP in Children (Version 2023). The diagnosis of pediatric mild MPP adhered to the following criteria^34^: age under 18 years; positive for MP nucleic acids and antibodies; clinical presentation of pneumonia (fever, cough, pulmonary signs); and lung infiltration evident on chest X‐ray or CT scan. Patients with mild MPP typically experience a disease course of 7–10 days and generally have a good prognosis without sequelae. Severe MPP presents with greater severity than mild MPP and meets at least one of the following criteria: (1) continuous high fever (≥39°C) for ≥ 5 days or fever for ≥ 7 days, without a decreasing trend in peak body temperature; (2) presence of wheezing, shortness of breath, difficulty breathing, chest pain, hemoptysis, or other symptoms indicative of severe lesions, concomitant plastic bronchitis, asthma exacerbations, pleural effusion, or pulmonary embolism; (3) Extrapulmonary complications without meeting critical care criteria; (4) pulse oxygen saturation ≤ 0.93 at rest while inhaling air; (5) imaging findings demonstrating one of the following criteria: (a) single lung lobe involvement ≥2/3 with uniform, high‐density consolidation or high‐density consolidation in two or more lung lobes (regardless of the affected area), potentially accompanied by moderate to large pleural effusion or localized bronchiolitis; (b) diffuse single lung or bilateral involvement ≥ 4/5 lobes with potential bronchiolitis, bronchitis, mucus plug formation and atelectasis; (6) progressive worsening of clinical symptoms and imaging evidence of lesion progression exceeding 50% within 24–48 h. Clinical features and laboratory findings used for disease severity classification are detailed in Supplementary Table 1.

### 4.3 scRNA sequencing

All BALF samples were collected during the acute stage of the MPP, as this stage provides valuable insights into the immune response during the initial and most severe phase of the disease. Samples were transported to the laboratory within 1 hour of collection. Cell suspensions were sequentially filtered using a 40 μm filter (Cat^#^ BS‐40‐C, Biosharp) and centrifuged at 500×g for 5 min at 4°C. Red blood cells were lysed using a 10‐fold volume of 1×Red Blood Cell Lysis Solution (Cat^#^ 30‐098‐462, MiltenyiBiotec). Dead cells are removed using the Dead Cell Removal Kit (Cat^#^ 130‐090‐101, MiltenyiBiotec) according to the manufacturer's instructions.

Cell viability, concentration, and clumping rate were evaluated using an acridine orange/propidium iodide (AOPI) stain (Cat^#^ F23001, Logos Biosystems) and a LUNA‐FLTM Automated Fluorescence Cell Counter (Cat^#^ L20001, Logos Biosystems). Samples were then centrifuged and re‐suspended in an appropriate volume of phosphate‐buffered saline (PBS) supplemented with 0.04% bovine serum albumin (BSA) and 1U/μL RNase inhibitor to achieve a final concentration of 700−1200 viable cells/μL. Samples were processed using the Chromium Next GEM Single Cell 5′ Kit v2 (Cat^#^ PN‐1000263, 10Xgenomics) and Chip K (Cat^#^ PN‐1000186, 10Xgenomics) on the Chromium Single Cell Controller (Cat^#^ PN‐1000204, 10XGenomics).

The scRNA‐seq library was prepared according to the manufacturer's instructions (CG000331, 10XGenomics). Library quality was assessed using the LabChip GX Touch Nucleic Acid Analyzer (Caliper Lifesciences) and StepOnePlus Real‐Time PCR System (Applied Biosystems) to confirm sequencing readiness. Libraries were sequenced on the Illumina NovaSeq6000 platform (Illumina) using a paired‐end 150 bp strategy with dual indexing and a minimum sequencing depth of 20,000 read pairs per cell.

### 4.4 Data analysis

Data generated by scRNA‐seq data were analyzed as previously described.^10^, ^12^ In brief, a merged, filtered gene expression matrix was generated for the 37 BALF samples using Kallisto/bustools (kb v0.24.4) and the ad.concat function in anndata (ad) (v0.7.6). Utilizing Scanpy (sc; v1.9.2), doublets and low‐quality cells were removed, and library size was normalized to 10,000 reads per cell. A consensus set of the top 1500 highly‐variable genes (HVGs) exhibiting significant cell‐to‐cell variation was determined.^12^ Data were integrated using principal component analysis (PCA) to reduce the dimensionality to 20 PCA components, followed by batch effect correction using the Harmony algorithm.^35^ Unsupervised clustering of single‐cell data was performed using the Louvain algorithm.^36^, ^37^

### 4.5 Cell clustering and annotations

Two rounds of unsupervised cell clustering were performed using the sc.tl.louvain function based on the neighborhood relationships. The first round of analysis used a Louvain resolution of 2.0 to identify and classify nine major cell types: CD4^+^ T cells (CD4), CD8^+^ T cells (CD8), plasma B cells, B cells, NK cells (natural killer cells), γδ T cells, epithelial cells (Epi), neutrophils (Neu), and macrophages (Macro).

Sub‐cluster within these major cell types representing distinct immune cell lineages were manually confirmed by examining canonical marker gene expression (Table S2). Signature genes for each cluster using the sc.tl.rank_genes_groups function and manually cross‐referenced with canonical marker genes for cluster annotation (Table S2).

### 4.6 Identifying changes in immune cell proportions

The distribution of each immune cell type or subtype across different diverse disease conditions was calculated, affirming their statistical significance. To determine tissue‐specific immune cell enrichment, the ratio of observed to expected cell counts (R_O/E_) was calculated for each cluster within each disease condition. Expected cell counts were determined using a Chi‐square test, assuming a null hypothesis of no disease‐specific enrichment. A R_O/E_ value greater than 1 indicates the enrichment of a specific immune cell cluster within a particular tissue. This approach, as previously described,^10^, ^12^ allowed for the identification of cell types exhibiting non‐random disease distribution patterns. The effects of different disease conditions and their potential interactions on the proportion of each cell type or subtype were analyzed using multivariate analysis of variance (ANOVA).^12^

### 4.7 Determining cell state scores

Predefined gene sets were used to measure the activation level or physiological activity of different cell types and subtypes. Gene sets associated with pro‐inflammatory cytokines and inflammatory responses were extracted from published literature (Table S6).^10^ Gene sets related to naïve state, exhausted, cytotoxic, regulatory effector, and IFN‐response, and other states were obtained from previous reports.^12^, ^29^ Cell state scores were calculated as the mean expression of genes within the predefined gene set divided by the mean expression of the reference genes using the sc.tl.score_genes function. The Kruskal‐Wallis Test with Bonferroni correction was used to determine the statistical significance of cell state scores when comparing different disease conditions.

### 4.8 Plasma cytokine assays and flow cytometric analysis

Plasma cytokine levels were measured using the Th1/Th2 34‐plex human ProcartaPlex kit (Thermo Fisher Scientific) according to the manufacturer's instructions and as previously described.^38^ Flow cytometric analysis was conducted as previously described and according to the manufacturer's instructions.^29^

### 4.9 Statistical analysis

Statistical analysis and data visualization were performed using Python and R. Statistical significance is indicated as follows: ns, *p* > 0.05; *, *p* ≤ 0.05; **, *p* ≤ 0.01; ***, *p* ≤ 0.001; ****, and *p* ≤ 0.0001

### 4.10 Code availability

The data and customized scripts utilized for data analysis can be shared upon a reasonable request.

## AUTHOR CONTRIBUTIONS

Yi Wang and Guogang Xu conceived the study; Yi Wang, Lin Gong, and Chengliang Zhu designed the study; Yi Wang, Guogang Xu, Lin Gong, and Chengliang Zhu supervised this project; Xiantao Shen, Zhengjiang Jin, Xiaomin Chen, Zhenhui Wang, Lu Yi, and Yangwei Ou contributed the experiments, reagents, and materials. Yi Wang, Xiantao Shen, and Lin Gong founded the study; Yi Wang contributed to the analysis tools. Yi Wang performed the software; Xiantao Shen, Zhengjiang Jin, Xiaomin Chen, Zhenhui Wang, Lu Yi, Yangwei Ou, Guogang Xu, Chengliang Zhu, Lin Gong, and Yi Wang analyzed the data; Yi Wang drafted the original paper; Yi Wang and Guogang Xu revised and edited this paper; Guogang Xu, Chengliang Zhu, Lin Gong, and Yi Wang reviewed the paper. All authors have read and approved the final manuscript.

## CONFLICT OF INTEREST STATEMENT

The authors declare no conflict of interest.

## ETHICS STATEMENT

The ethical approval for this study was obtained from the Ethics Committee of the Maternal and Child Health Hospital of Hubei Province (Ethical approval NO.2023IEC122).

## PATIENT CONSENT STATEMENT

Written informed consent was acquired from each participant.

## Supporting information



Supporting Information

Supporting Information

## Data Availability

The data that support the findings of this study are openly available in the China National Center for Bioinformation at https://ngdc.cncb.ac.cn/omix/release/OMIX007208; reference number OMIX007208. The data reported in this paper have been deposited in the OMIX, China National Center for Bioinformation/Beijing Institute of Genomics, Chinese Academy of Sciences (https://ngdc.cncb.ac.cn/omix; accession no. OMIX007208).
