# Supplementary material for: Single‐cell transcriptome atlas revealed bronchoalveolar immune features related to disease severity in pediatric Mycoplasma pneumoniae pneumonia
Source: MedComm (2020). 2024 Oct 13;5(10):e748. doi: 10.1002/mco2.748 (PMC11471001; doi:10.1002/mco2.748)
Supplement: Supplementary file 1 — Supporting Information [file MCO2-5-e748-s001.pdf]

Supplementary Information

Single cell transcriptome atlas revealed bronchoalveolar immune features related to disease severity in pediatric *Mycoplasma pneumoniae* pneumonia

Xiantao Shen [1,#], Zhengjiang Jin [2,#], Xiaomin Chen [3,#], Zhenhui Wang [2], Lu Yi [2], Yangwei Ou [4], Lin Gong [1,3,*] , Chengliang Zhu [5,*], Guogang Xu [6,*],and Yi Wang [7,*]

[1] State Key Laboratory of Environment Health (Incubation), Key Laboratory of Environment and Health, Ministry of Education, Key Laboratory of Environment and Health (Wuhan), Ministry of Environmental Protection, School of Public Health, Tongji Medical College, Huazhong University of Science and Technology, Wuhan, Hubei, 430030, China.

[2] Department of Clinical Laboratory, Maternal and Child Health Hospital of Hubei Province, Tongji Medical College, Huazhong University of Science and Technology, Wuhan, Hubei, 430070, China

[3] Department of Disinfection and Pest Control, Wuhan Center for Disease Control & Prevention, Wuhan, Hubei, 430000, China.

[4] Department of Radiology, Maternal and Child Health Hospital of Hubei Province, Tongji Medical College, Huazhong University of Science and Technology, Wuhan, 430070, China,

[5] Department of Clinical Laboratory, Institute of Translational Medicine, Renmin Hospital of Wuhan University, Wuhan, Hubei, 430060, China

[6] Health Management Institute, The Second Medical Center & National Clinical Research Center for Geriatric Diseases, Chinese PLA General Hospital, 28 Fuxing Road, Beijing, 100853, China.

[7] Experimental Research Center, Capital Institute of Pediatrics, Beijing, 100020, China.

[#] These authors contributed equally.

*Correspondence: Prof. Yi Wang, wildwolf0101@163.com, Prof. Guoang Xu, gxu@301hospital.org, Prof. Chengliang Zhu, zhuchengliang@whu.edu.cn, Dr. Lin Gong, gonglin_1@163.com.

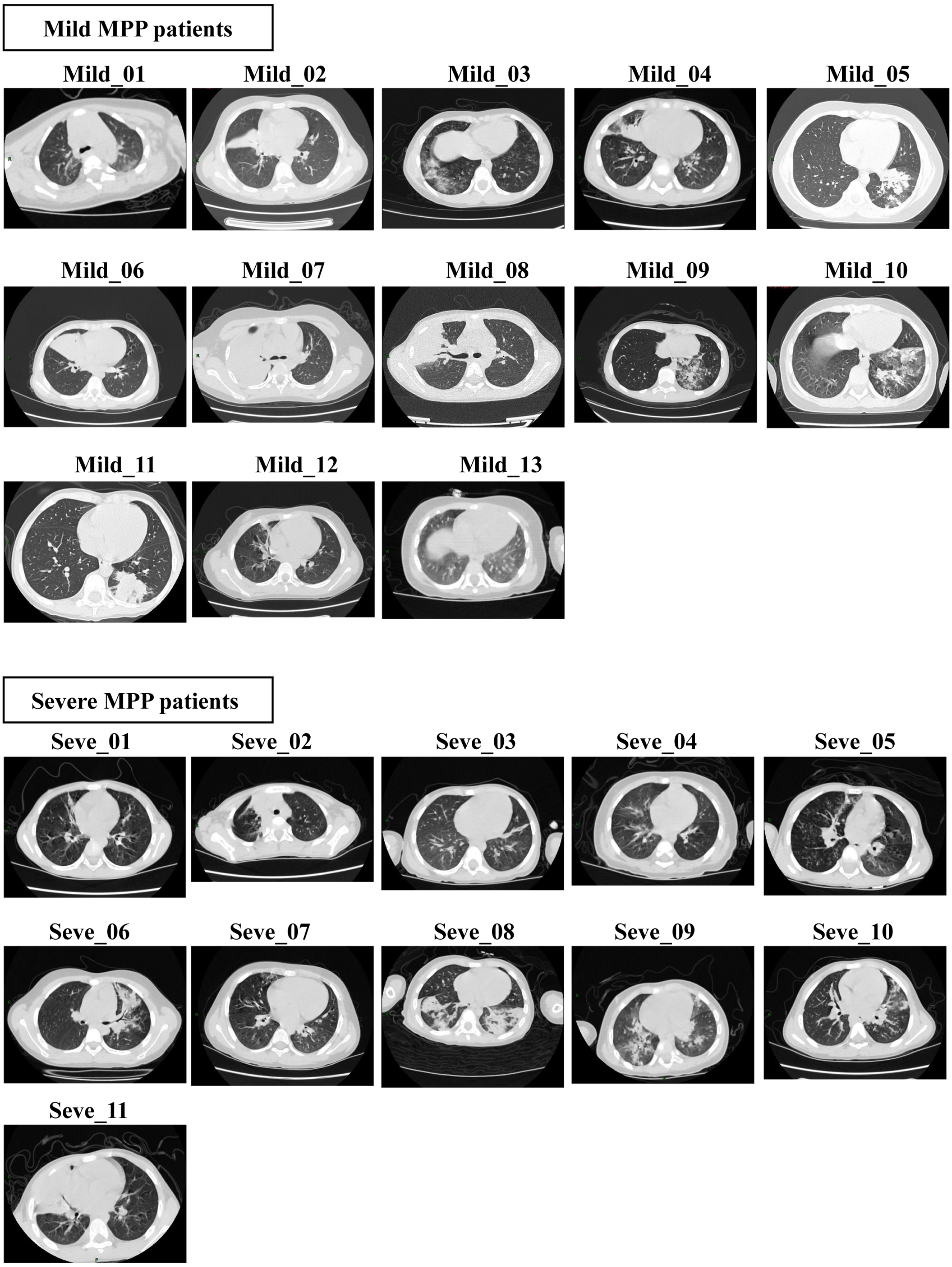

Supplementary Fig. 1 Chest CT images from mild and severe cases.

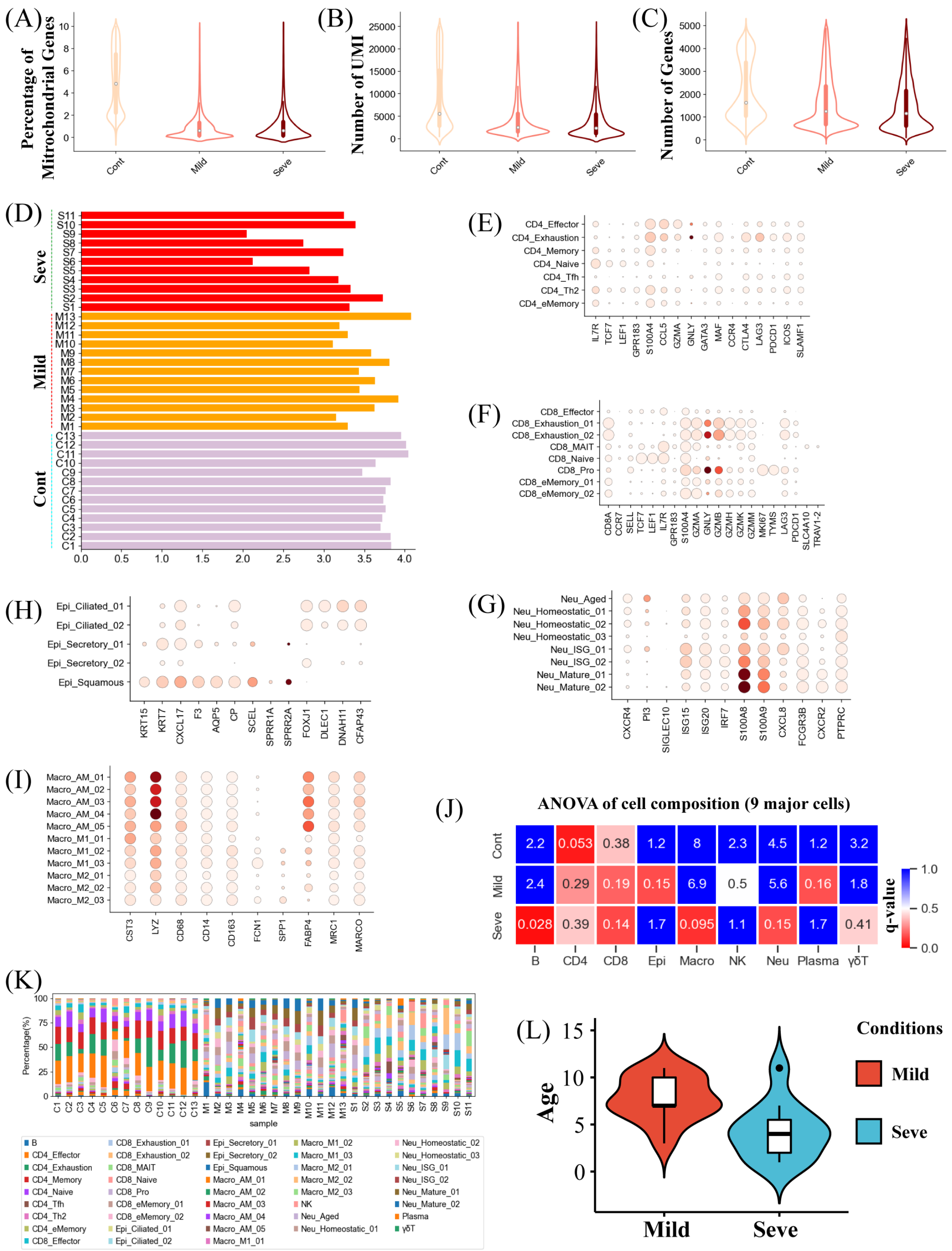

Supplementary Fig 2. Detailed data output and visualization of single-cell transcriptional profiling of BALFs from 37 subjects, related to figure 1. A-C. Percentage of mitochondrial transcripts per cell (A), distribution of unique molecular identifier (UMI) counts per cell (B) and the gene counts per cell (C) detected for cells in each disease group. D. Box plots illustrating the $\log_{10}$ transformed number of cells for each sample. E-I. Dot plots of selected marker genes (Rows) for cell subsets (Columns) within each cell lineage, including $CD4^{+}$T (E), $CD8^{+}$T (F), neutrophil (G),

epithelial (H) and macrophage (I) cell subsets. J. Heatmap showing the association between cell composition and disease types. The color represents ANOVA q values. K. Stacked bar plot showing the relative proportion of cell subtypes derived from Cont, Mild and Seve conditions. L. Violin plots depict the age distributions of pediatric patients with *Mycoplasma pneumoniae* pneumonia stratified by disease severity. No statistically significant difference in age was observed between patients with mild and severe *Mycoplasma pneumoniae* pneumonia.

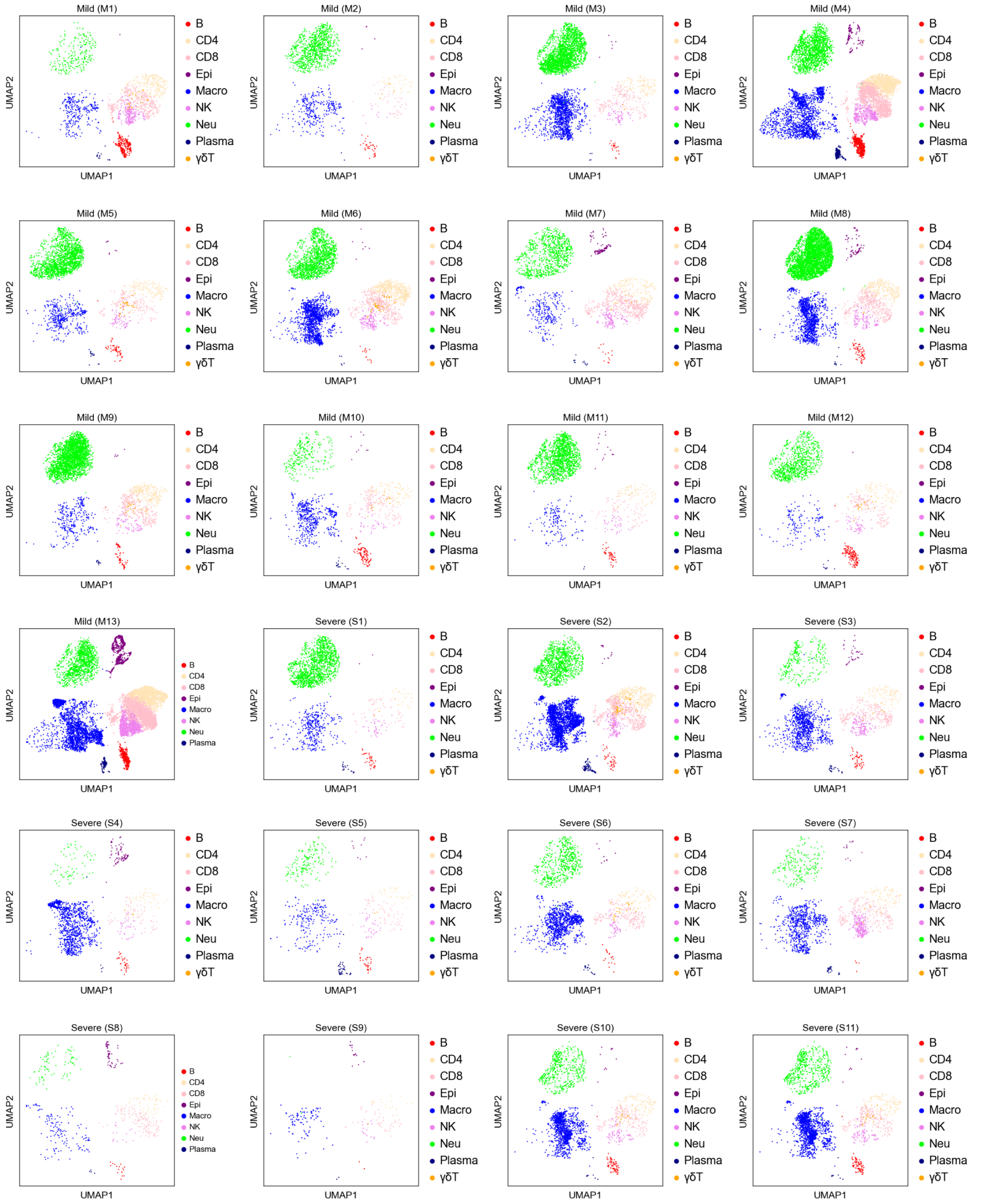

Supplementary Fig 3. UMAP projection of the 24 MPP patients. Colored based on the 9 major cell types.

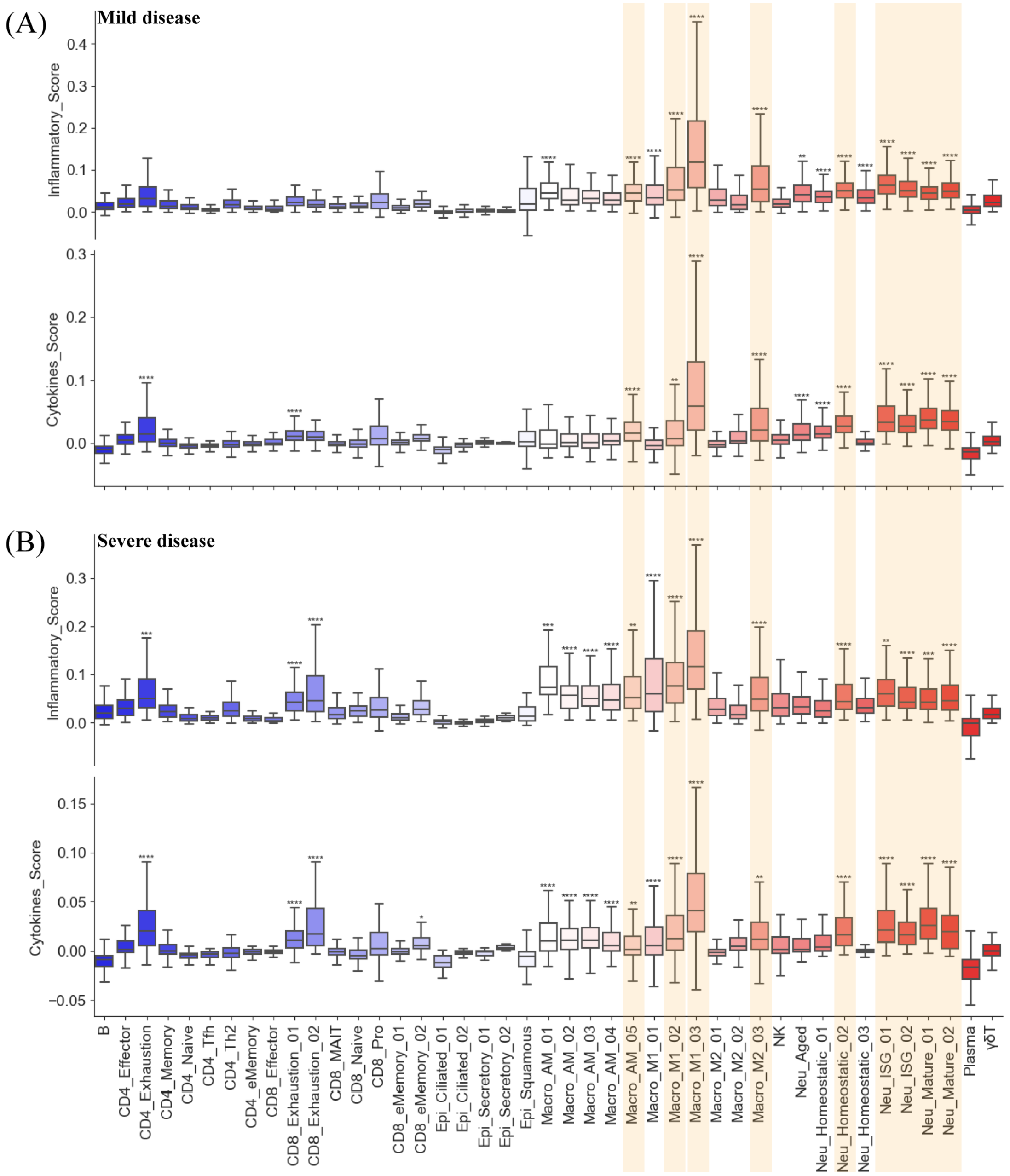

Supplementary Fig 4. Identification of hyper-inflammatory subtypes associated with potential inflammatory response in BALFs, related to Figure 2. A. Boxplots of the inflammatory score (top panel) and cytokine score (bottom panel) of cell subtypes in mild cases. Significance was evaluated using the Kruskal-Wallis test with Bonferroni correction. ****$p < 0.0001$. B. Boxplots of the inflammatory score (top panel) and cytokine score (bottom panel) of cell subtypes in severe cases. Significance was evaluated using the Kruskal-Wallis test with Bonferroni correction. ****$p < 0.0001$.

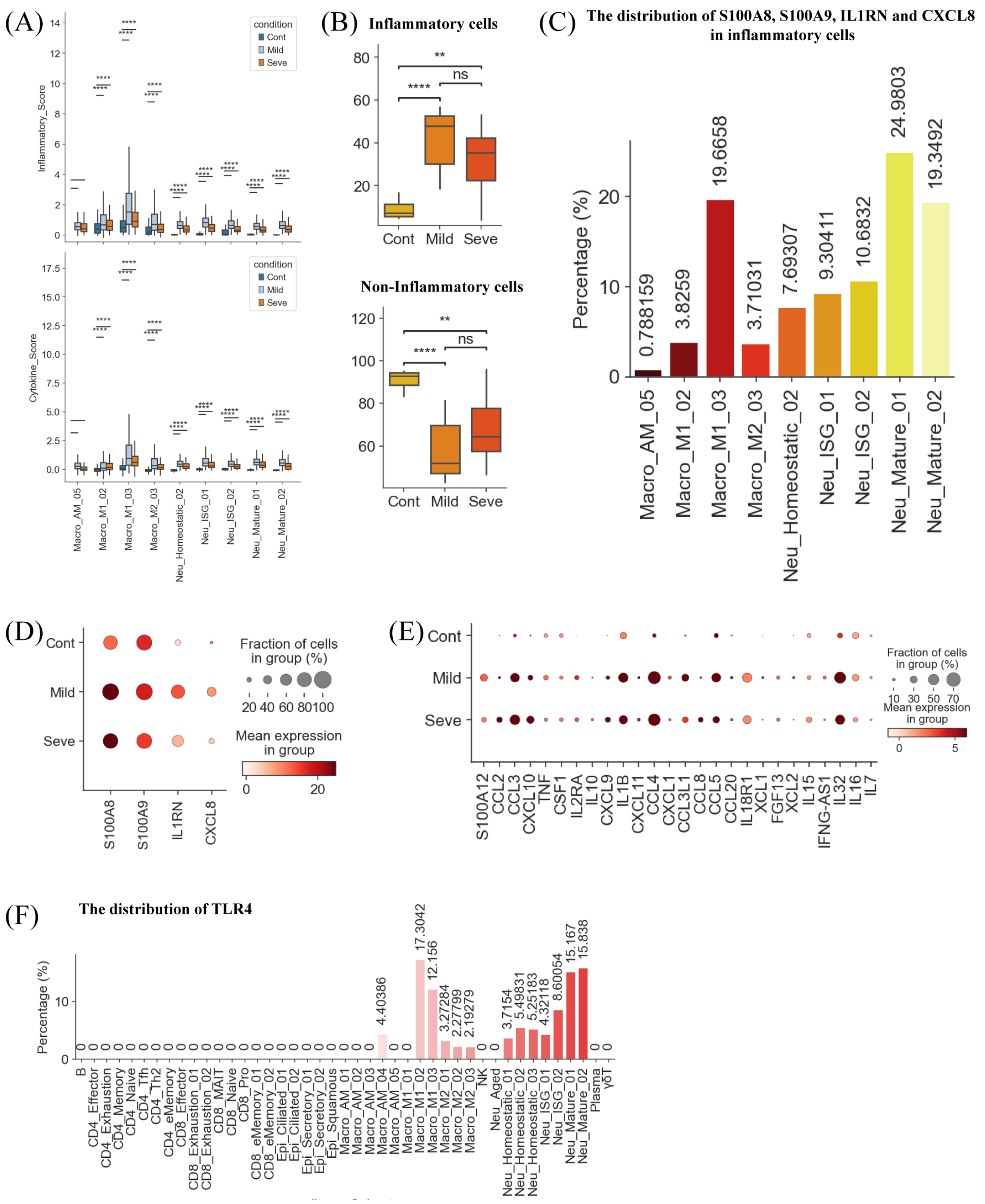

Supplementary Fig 5. Details of hyper-inflammatory subtypes associated with potential inflammatory response in BALFs, related to Figure 2

A. Box plots of the expression levels of inflammatory score (top panel) and cytokine score (bottom panel) derived from healthy donors, mild cases and severe cases. Horizontal lines represent median values, with whiskers extending to the farthest data point within a maximum of 1.5 × interquartile range. Significance was evaluated using the Kruskal-Wallis test with Bonferroni correction (*p<0.05, **p<0.01, ***p<0.001, ****p<0.0001, ^{ns}p>0.05). B. Box plots showing the proportion of inflammatory cell types (Left panel) and other cell types (Right panel) across

conditions. Significance was evaluated using the Kruskal-Wallis test with Bonferroni correction (*p<0.05, **p<0.01, ***p<0.001, ****p<0.0001, ^{ns}p>0.05). C. Bar plots showing the distribution of S100A8/A9, IL1RN and CXCL8 in inflammatory cells. D. Dot plots showing the selected genes (S100A8/A9, IL1RN and CXCL8) in inflammatory cells across three conditions. E. Dot plots showing the selected genes in inflammatory cells across three conditions. F. Bar chart depicting the relative contribution of the *TLR4* in cell subtypes.

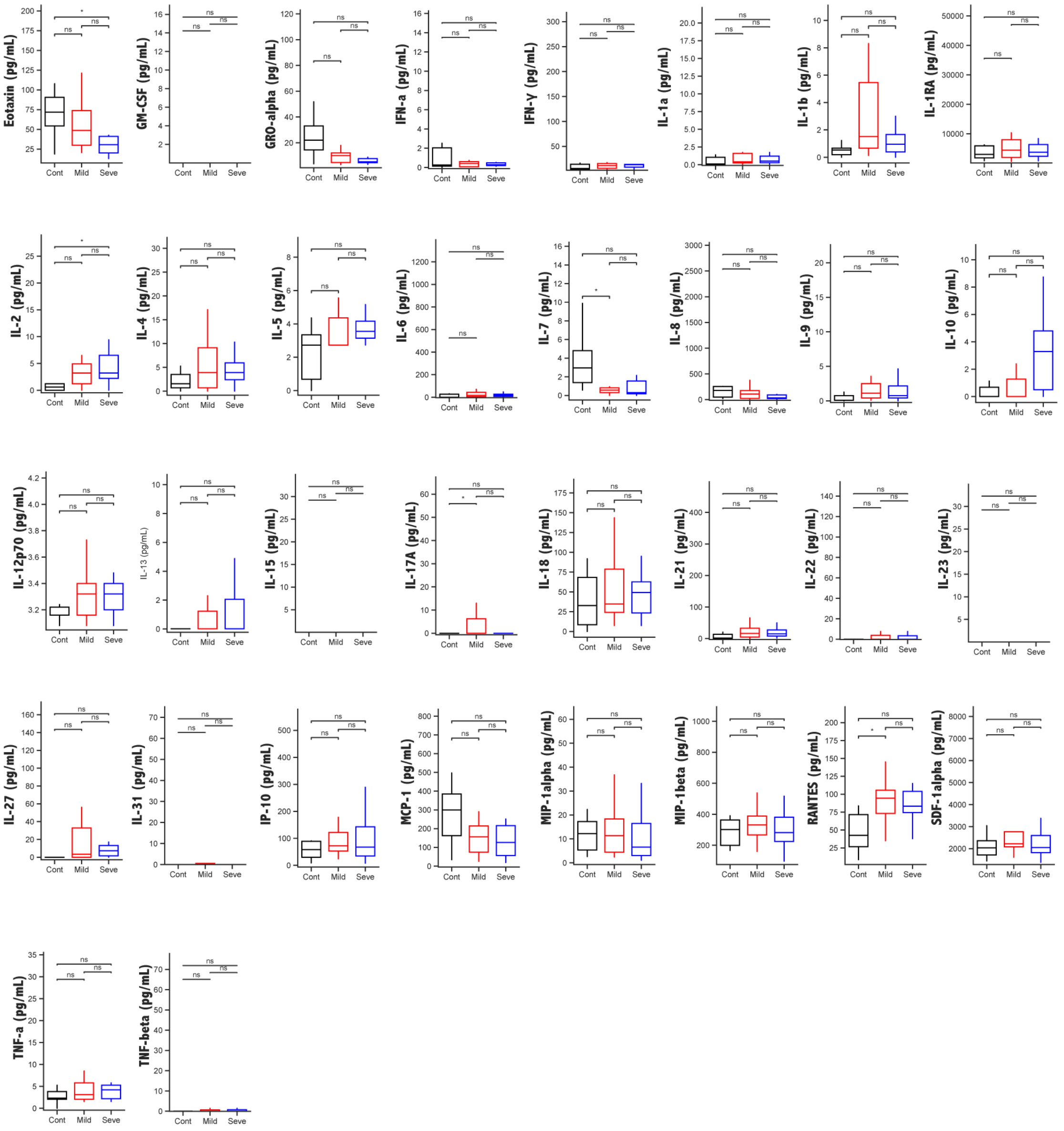

Supplementary Fig 6. The levels of 32 cytokines in each group. Significant differences were determined by Kruskal-Wallis test with Bonferroni correction (*p<0.05, **p<0.01, ***p<0.001, ****p<0.0001, ^{ns}p>0.05).

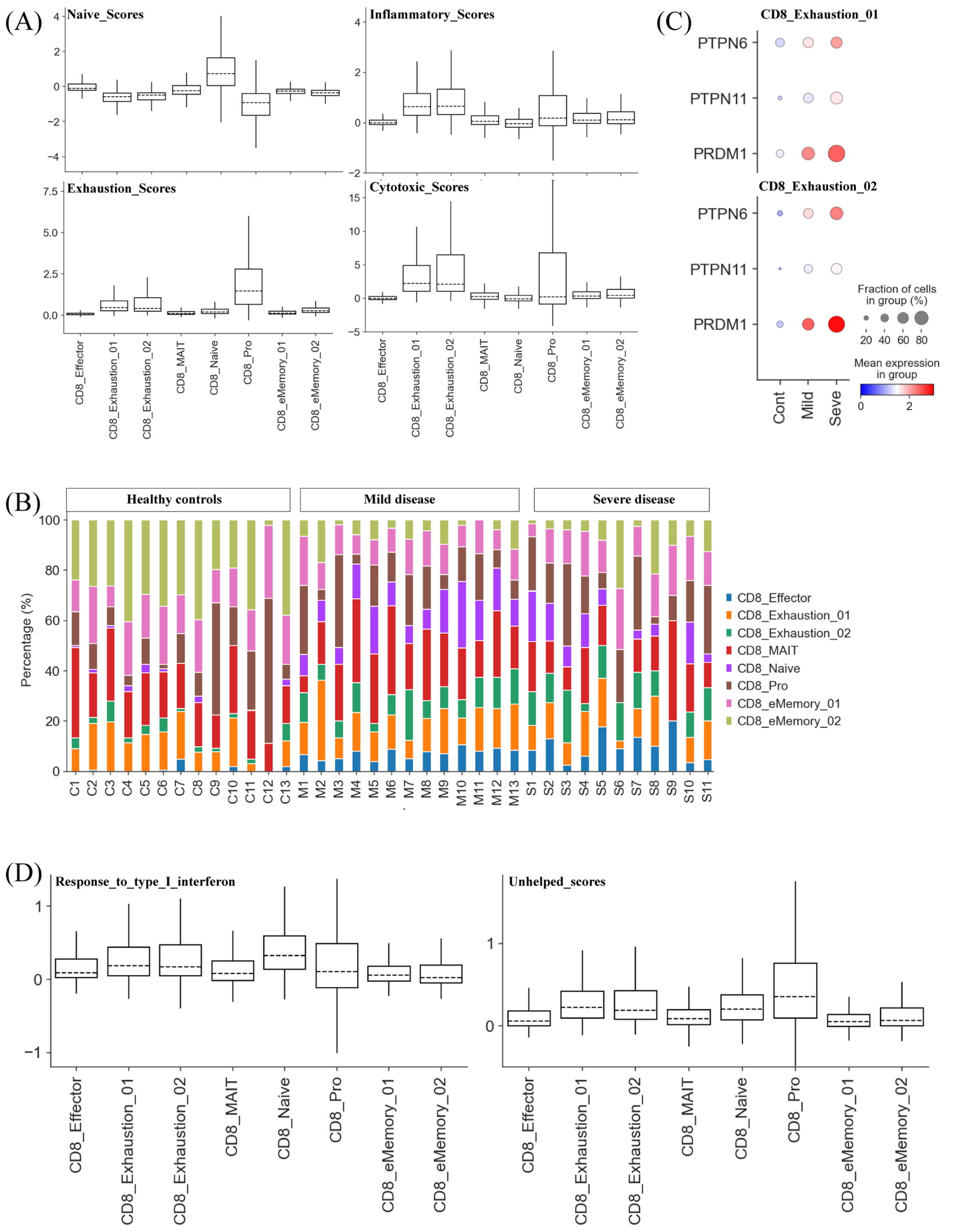

Supplementary Fig 7. Characterization of gene expression differences in CD8^{+}T cells across conditions, related to Figure 3. A. Box plot showing the indicated functional scores of CD8^{+}T cell subtypes. B. Stacked bar plot showing the relative proportion of 8 CD8^{+}T cell subtypes derived from Cont, Mild and Seve conditions. C. Dot plots showing the expression of selected genes in CD8_Exhaustion_01 and CD8_Exhaustion_02. D. Box plot showing the indicated functional scores of CD8^{+}T cell subtypes.

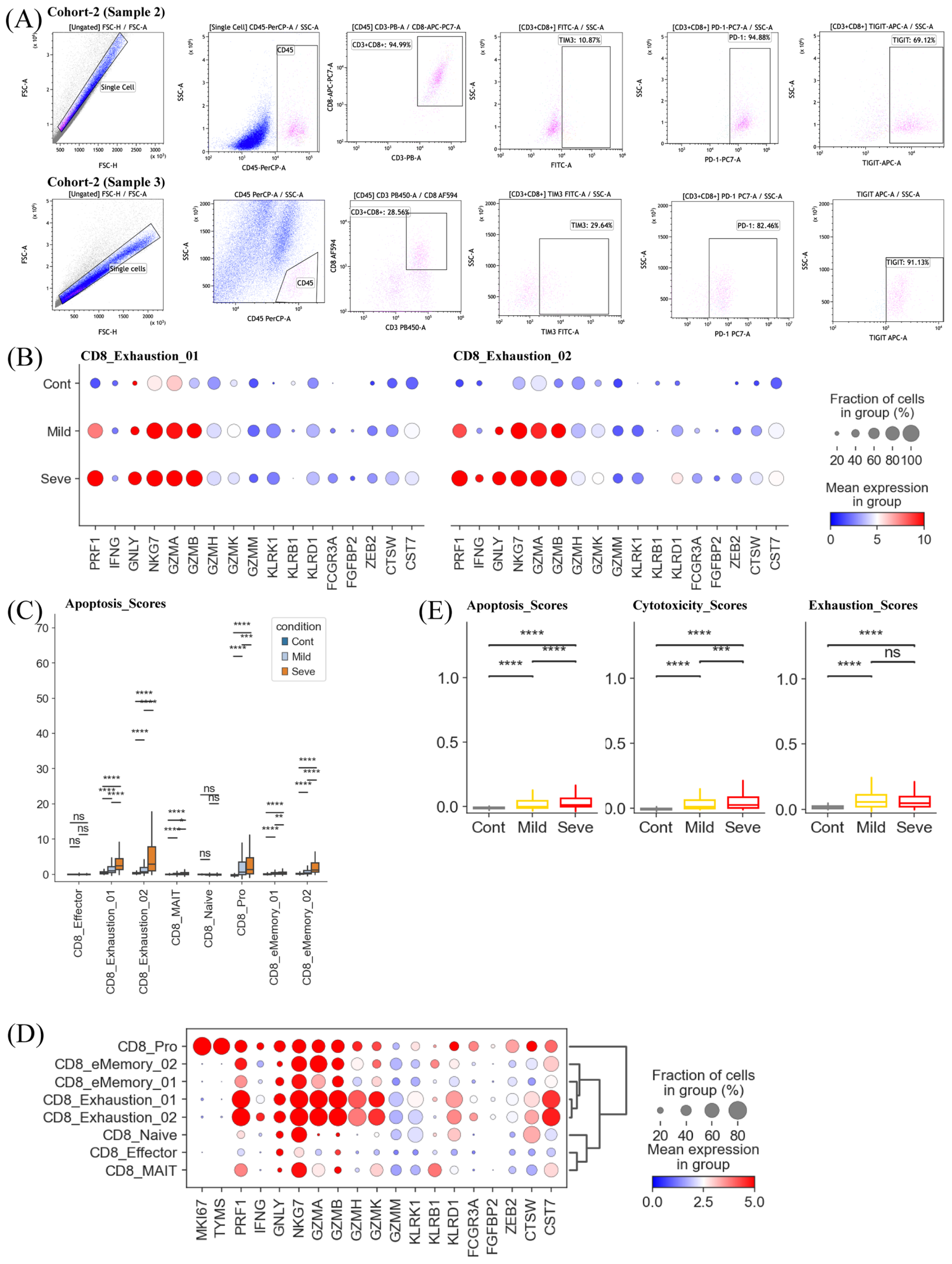

Supplementary Fig 8. Characterization of gene expression differences in CD8^{+}T cells across conditions, related to Figure 3. A. Flow cytometry plots showing gating strategy and typical exhausted molecules in CD8^{+}T cells from severe cases (Cohort 2-Sample 1). B. Dot plots showing the expression of selected genes in CD8_Exhaustion_01 and CD8_Exhaustion_02. C. Boxplots of the apoptosis score of CD8^{+} T cell subtypes. Significance was evaluated using the Kruskal-Wallis test with

Bonferroni correction. ****$p < 0.0001$. D. Dot plots showing the expression of selected genes in CD8_Exhaustion_01 and CD8_Exhaustion_02. E. Box plot showing the apoptosis score, cytotoxicity score and exhaustion scores in different CD8^{+}T cell subtypes.

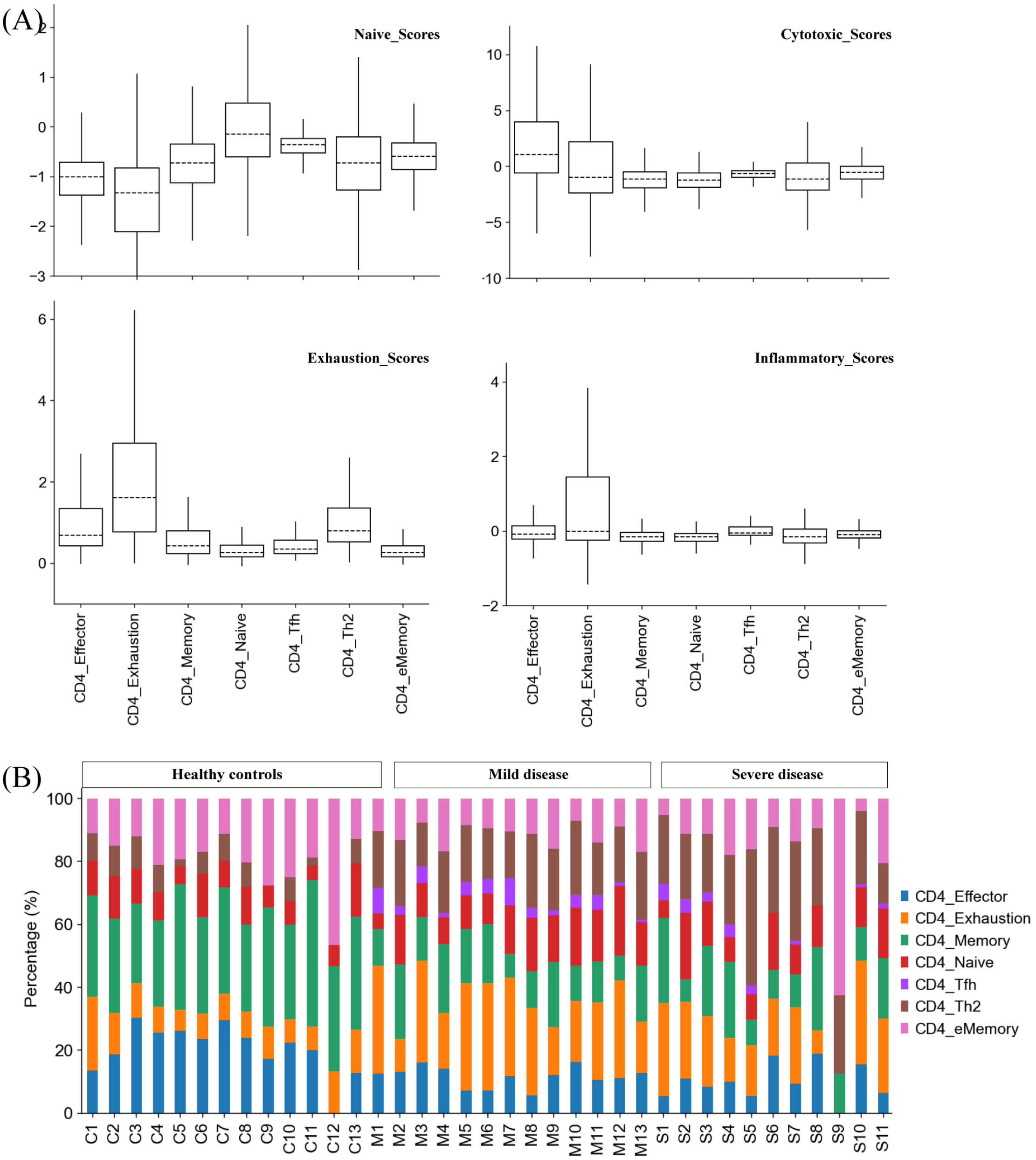

Supplementary Fig 9. Characterization of gene expression differences in CD4^{+}T cells across conditions, related to Figure 4. A. Box plot showing the indicated functional scores of CD4^{+}T cell subtypes. B. Stacked bar plot showing the relative proportion of 7 CD4^{+}T cell subtypes derived from Cont, Mild and Seve conditions.

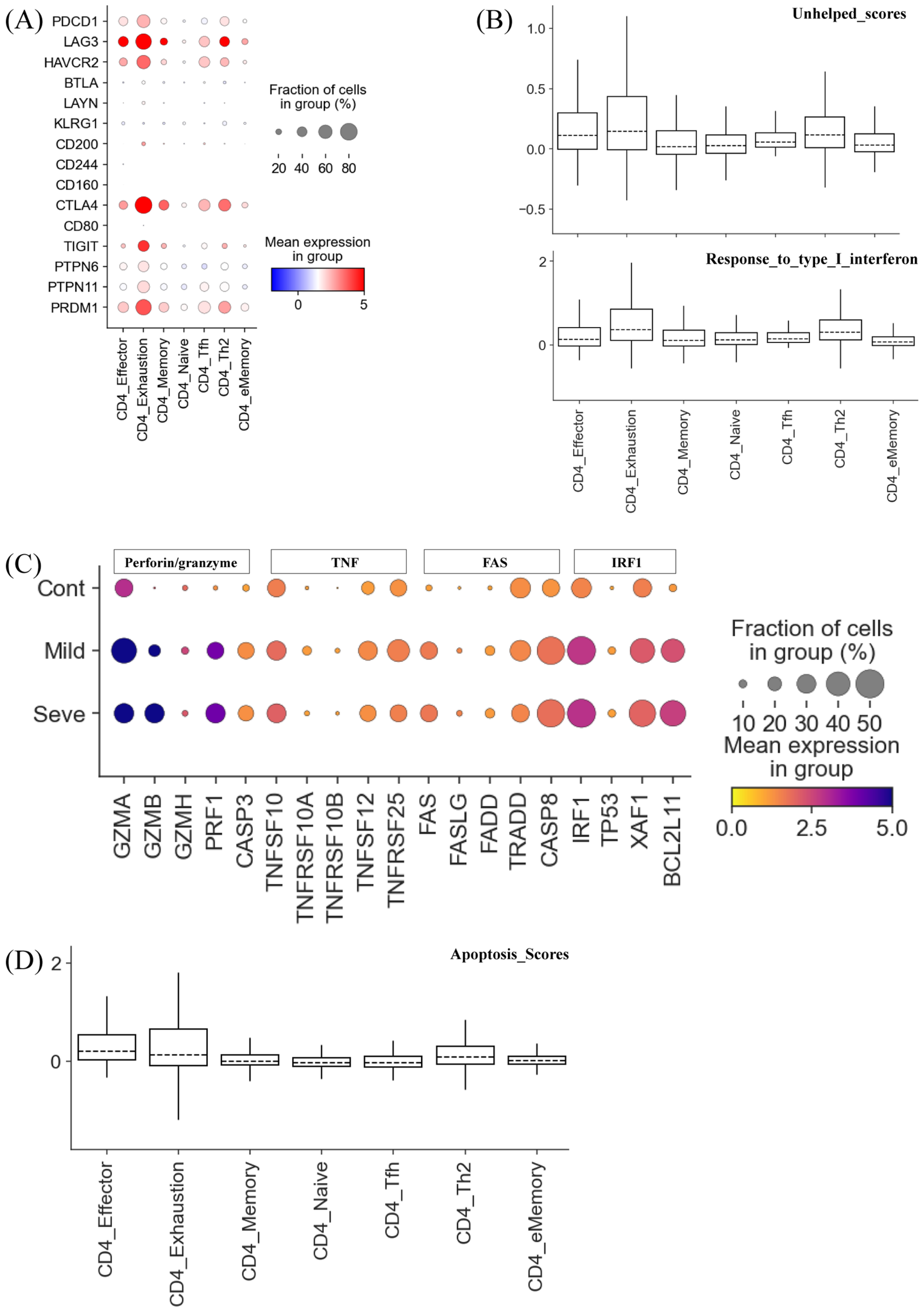

Supplementary Fig 10. Characterization of gene expression differences in CD4$^+$T cells across conditions, related to Figure 4. A. Dot plots showing the expression of exhaustion-related genes in CD4$^+$T-cell subsets. B. Box plot showing the indicated functional scores of CD4$^+$T cell subtypes. C. Dot plots showing the expression of

apoptosis-related genes in CD4$^+$T-cells across different conditions. D. Box plot showing the indicated apoptosis scores of CD4$^+$T cell subtypes.

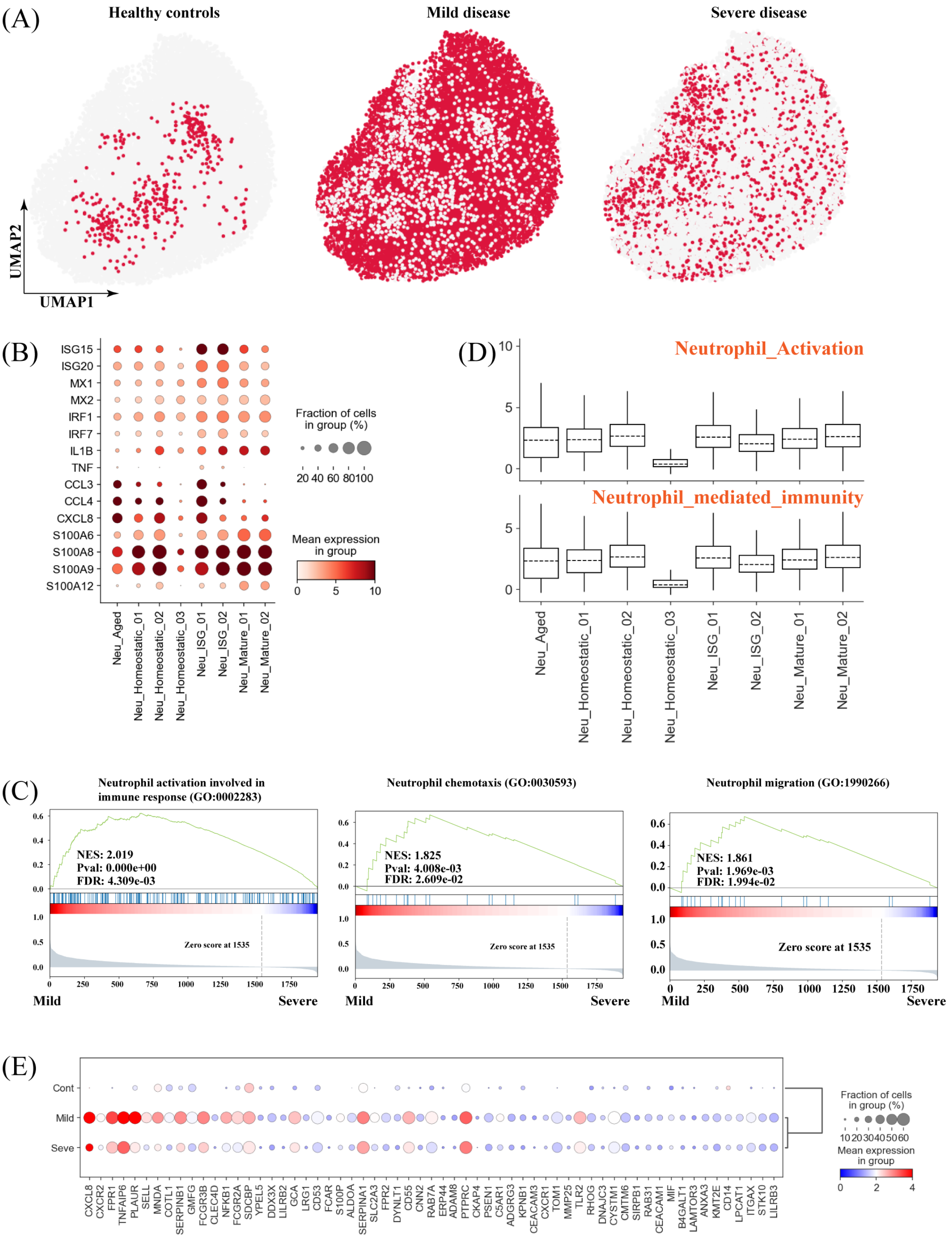

Supplementary Fig 11. Characterization of gene expression differences in neutrophil across conditions, related to Figure 5. A. UMAP projections of neutrophils splitted by condition. B. Dot plots showing the expression of selected genes in neutrophil subsets. C. Gene Set Enrichment Analysis of the sets compared between mild and severe groups with the neutrophil activation, chemotaxis and migration gene sets. D. Box plot showing the indicated functional scores of neutrophil

subtypes. E. Dot plots showing the expression of selected genes in neutrophils across different conditions.

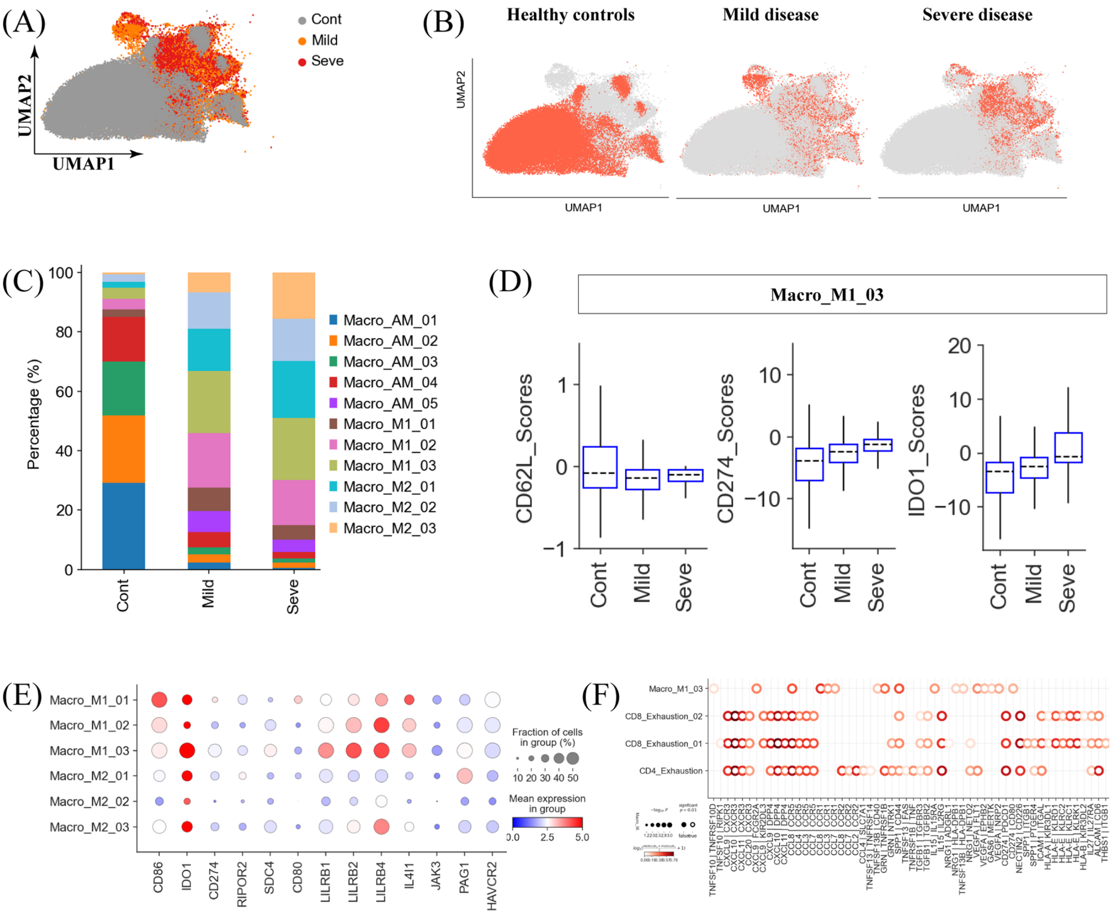

Supplementary Fig 12. Characterization of gene expression differences in macrophages across conditions, related to Figure 6. A. UMAP projections of macrophage. Colored based on the 3 conditions. B. UMAP projections of macrophage splitted by conditions. C. Stacked bar plot showing the relative proportion of 11 macrophage subtypes derived from Cont, Mild and Seve conditions. D. Box plot showing the indicated gene scores of 3 conditions. E. Dot plots showing the expression of selected genes in M1-/M2-like macrophage subsets. F. Dot plot of the interactions between Macro_M1_03 and exhausted T cell types in severe MPP patients. P values are indicated by the circle sizes, as shown in the scale on the right.
